# Feeding Behaviours in Families With Children or Young People With Autism: A Systematic Review

**DOI:** 10.1111/nbu.70066

**Published:** 2026-08-18

**Authors:** Emma Tuschick, Jo Smith, Benjamin Harrison, Matthew Youngman, Lee Copping, Emma L. Giles

**Affiliations:** ^1^ School of Health and Life Sciences Teesside University Middlesbrough UK; ^2^ School of Social Sciences, Humanities and Law Teesside University Middlesbrough UK

**Keywords:** autism spectrum conditions, children, feeding behaviours, food refusal, food selectivity, sensory sensitivity, systematic review, young people

## Abstract

Children and young people with autism often experience feeding difficulties and are at increased risk of food insecurity. However, the interplay between these challenges remains underexplored. This systematic review aimed to synthesise existing evidence on the prevalence and characteristics of feeding behaviours in families with children with autism, alongside associated factors such as sensory sensitivity and mealtime dynamics. Nine databases identified 399 records. Following screening, 88 original empirical research studies (including quantitative, qualitative and mixed‐methods designs) were included. Studies were included if they investigated children and young people with autism under 25 years and focused on feeding behaviours. Reviews, meta‐analyses, editorials, protocols and grey literature were excluded. Included studies were critically appraised and findings were synthesised using narrative analysis. The analysis highlighted widespread food selectivity, sensory sensitivities and restrictive eating patterns, contributing to nutritional deficiencies and parental stress. Children with autism commonly preferred starchy foods and snack foods, alongside reduced consumption of protein‐rich foods, with mealtime challenges including refusal and social disruption. Sensory processing difficulties and autism severity were strongly linked to feeding behaviours. Feeding difficulties are prevalent among children with autism and are shaped by sensory sensitivities and family dynamics. Tailored interventions, standardised assessment tools and further research into the social and nutritional implications of feeding challenges are needed to better support children with autism and their families.

## 1 Introduction

Autism is a lifelong neurodevelopmental condition that affects how individuals communicate, interact socially, process information and behave. Although diagnosis can occur at any age, autism is considered a developmental disorder because signs typically emerge within the first two years of life (National Institute of Mental Health 2022). Globally, around 1% of children are diagnosed with autism, with boys diagnosed approximately four times more often than girls (Drake Institute 2024). This gender disparity is partially attributed to diagnostic biases, as girls with autism may be more likely to exhibit internalised difficulties, develop compensatory social strategies and engage in social camouflaging that can mask autistic traits, resulting in delayed recognition and underdiagnosis (Drake Institute 2024).

Feeding behaviours refer to the actions and patterns individuals exhibit related to food intake (Petrovich 2019). These behaviours encompass a wide range of activities including food selection, portion control, eating pace, meal frequency and responsiveness to hunger and satiety cues (Oliveira et al. 2020). In the context of children with autism, atypical feeding behaviours may include food aversions, limited food preferences, ritualistic eating habits, food refusal, sensory sensitivities and difficulties with mealtime routines and social interactions surrounding food (Baraskewich et al. 2021; Adams 2022).

Research suggests that children with autism are approximately five times more likely to experience feeding difficulties than children without autism, with around 62% presenting with some form of feeding challenge (Sharp et al. 2013, 2014). Feeding behaviours in children with autism vary considerably in both presentation and severity, ranging from mild food preferences to severe food selectivity, food refusal and sensory‐related feeding difficulties. Although these behaviours are often associated with nutritional concerns, the evidence regarding their impact on weight status and dietary intake is mixed. Some studies have reported increased rates of obesity, eating disorders and nutritional deficiencies among children with autism (National Autistic Society 2024; Robea et al. 2020; Thom et al. 2022), whereas others have found limited or inconsistent associations between feeding behaviours and growth, weight status or overall nutrient intake. Emerging evidence (Li et al. 2024; Wenzell et al. 2024) also suggests that adverse nutritional outcomes may be concentrated within specific subgroups of children, such as those with more severe food selectivity, sensory sensitivities or co‐occurring feeding disorders, rather than being characteristic ofchildren with autism as a whole. Consequently, there remains considerable uncertainty regarding the prevalence, nature and consequences of feeding behaviours in this population. A clearer understanding of these relationships is important for informing clinical practice, identifying children most at risk of adverse outcomes and guiding the development of targeted interventions.

In the United Kingdom, it is estimated in 2024 that around 700 000 people are autistic, making up over 1% of the population (National Autistic Society 2024). Individuals with autism are reported to be 1.5 times more likely to experience food insecurity than the general population (Karpur et al. 2021). Food insecurity refers to limited or uncertain access to sufficient, safe and nutritious food and is often influenced by broader socioeconomic factors including household income, rising living costs and increased healthcare and educational expenses (Mccall and Starr 2016; Rogge and Janssen 2019). Although food insecurity and feeding difficulties are distinct concepts, they may interact in important ways within families of children with autism. Restricted eating patterns, food selectivity and sensory‐related feeding difficulties may increase financial and practical pressures by limiting the range of foods a child will accept, potentially leading to increased food waste and higher food costs. Conversely, food insecurity may exacerbate feeding difficulties when preferred foods are unavailable, routines are disrupted or parental stress increases. Together, these challenges may place additional strain on family well‐being and caregiving capacity, highlighting the need for a better understanding of how feeding behaviours and food insecurity intersect within this population (Hazzard et al. 2022).

Despite growing recognition of feeding difficulties in autistic populations, research remains limited, particularly in exploring the intersection between autism and feeding behaviours. Addressing this gap is essential to better support families navigating these challenges. Therefore, this systematic review aimed to synthesise the current evidence on feeding behaviours among children and young people with autism. Although feeding behaviours were the primary focus of the review, studies examining food insecurity were also included where they explored its relationship with feeding behaviours, nutritional intake or eating patterns. Food insecurity was conceptualised as a contextual factor that may influence, or be influenced by, feeding difficulties rather than as a separate outcome. Specifically, the review sought to examine: (1) food selectivity, food refusal, sensory‐related feeding behaviours and other feeding difficulties; (2) dietary diversity, nutritional status, weight‐related outcomes and food insecurity were examined alongside feeding behaviours; (3) demographic, environmental and sensory factors associated with feeding difficulties and (4) interventions designed to improve feeding behaviours and mealtime experiences among children with autism.

## 2 Methods

This review adheres to the PRISMA 2020 (Preferred Reporting Items for Systematic Reviews and Meta‐Analyses) guidelines to ensure transparent and comprehensive reporting (Shamseer et al. 2015). The review protocol was not registered on PROSPERO, as the work originated from a master's dissertation project. However, the review has since been refined and the literature searches have been updated and repeated accordingly.

### 2.1 Search Strategy

Initial searches were conducted in June 2023, with subsequent updates in December 2023 and February 2025. Eight academic databases were searched: Web of Science, Scopus, Medline, APA Psych, PsychInfo, CINAHL, PubMed and ASSIA. Additionally, Google Scholar was searched, with the first 10 pages of results screened for relevance due to their likelihood of containing high‐quality sources (Haddaway et al. 2015). All identified records were imported into Rayyan (Ouzzani et al. 2016), where duplicates were automatically detected and removed before screening commenced.

A structured search was carried out following the PICOS framework (Methley et al. 2014) to guide the identification of relevant studies for a mixed‐methods synthesis. The population of interest included children and young people under 25 years of age with diagnosed or self‐diagnosed autism spectrum conditions, as well as their families. The intervention/exposure focused on feeding behaviours, whereas comparators referred to neurotypical individuals without neurological or developmental conditions. The outcomes of interest were reports or measures of feeding behaviours captured in cross‐sectional, cohort or other quantitative studies. Outcomes included food selectivity, food refusal, food neophobia, dietary diversity, nutritional status, mealtime behaviours, sensory feeding difficulties, feeding intervention outcomes and food insecurity. Full database search strategies, including MeSH terms and keyword combinations, are provided in File S1. Eligible studies included peer‐reviewed quantitative, qualitative and mixed‐methods primary research articles. Reviews, meta‐analyses, conference abstracts, editorials, commentaries, protocols, dissertations and grey literature were excluded.

### 2.2 Eligibility Criteria

To be included, studies had to be primary research articles written in English, published in peer‐reviewed journals since 2013. The 2013 cut‐off was selected to ensure the review reflected contemporary evidence and current conceptualisations of autism and feeding behaviours, whereas maintaining a manageable and relevant evidence base. This period also captures research conducted following the publication and widespread adoption of DSM‐5 diagnostic criteria. Studies examining food insecurity were eligible only where food insecurity was analysed in relation to feeding behaviours, dietary intake, nutritional status or mealtime experiences among autistic children. Eligible studies were required to: (1) be primary empirical research; (2) be published in English in peer‐reviewed journals; (3) include children or young people with autism aged under 25 years and (4) examine feeding behaviours, eating patterns, nutritional intake, feeding difficulties, sensory‐related feeding issues, food selectivity, food refusal or food insecurity. Studies were excluded if they were reviews, meta‐analyses, editorials, conference abstracts, dissertations, protocols, commentaries, grey literature, or did not report feeding‐related outcomes for children with autism. No minimum sample size criterion was applied. This decision was made to ensure inclusion of the full range of available evidence, including qualitative studies, mixed‐methods research, pilot studies and intervention studies that may involve smaller samples but provide important insights into feeding behaviours among children and young people with autism.

### 2.3 Study Selection and Data Management

Following completion of the database searches, all identified records were exported into Rayyan and duplicates were removed before screening. The study selection process was conducted in two stages. First, titles and abstracts were screened against the predefined eligibility criteria. Two reviewers (E.T. and B.H.) independently screened the records, with each reviewer cross‐checking 20% of the other reviewer's decisions to assess consistency. Any differences in screening decisions were discussed between the two reviewers and resolved by returning to the eligibility criteria and reviewing the title and abstract again. Where uncertainty remained, the article was retained for full‐text screening. Inter‐rater reliability was high, with a Cohen's *κ* of 0.85 (93.2% agreement), indicating near‐perfect agreement and no disagreements required arbitration by a third reviewer.

Second, full‐text articles were retrieved for all records considered potentially eligible. One reviewer (ET) screened the full texts, whereas a second reviewer (B.H.) independently assessed 20% of the full‐text articles. Reasons for exclusion at the full‐text stage were recorded. Disagreements were resolved through discussion between E.T. and B.H., with decisions made by referring back to the inclusion and exclusion criteria. Arbitration by a third reviewer was planned if consensus could not be reached; however, this was not required. All included studies were then organised and stored in Microsoft Teams, and the final set of included articles was used for data extraction and quality appraisal. Agreement between reviewers at this stage was substantial (Cohen's *κ* = 0.62, 90% agreement), confirming the reliability of the screening process.

### 2.4 Data Extraction

A structured Microsoft Excel spreadsheet was created by two reviewers (E.T. and B.H.) to systematically extract relevant data from each study. The extracted fields included: author names, publication year, study location, research objectives, study design, methodology, sample size, diagnostic tools used to confirm autism, presence of co‐occurring conditions or disabilities, type of questionnaires utilised, details of any interventions, descriptions of feeding behaviours, participant demographics, outcome variables, key findings, conclusions and any recommendations provided by the authors.

### 2.5 Assessment of Quality

To evaluate the methodological quality of included studies, the reviewers (E.T. and B.H.) applied two standardised tools: the Appraisal Tool for Cross‐Sectional Studies (AXIS) (Downes et al. 2016) and the Mixed‐Methods Appraisal Tool (MMAT) (Hong et al. 2018).

The AXIS tool was used to assess the quality of cross‐sectional studies and consists of 20 items covering study design, reporting quality, sampling methods, measurement validity, risk of bias and the interpretation of results (Downes et al. 2016). Each item is rated as ‘Yes’, ‘No’ or ‘Do not know/comment’, allowing reviewers to identify specific methodological strengths and limitations.

The MMAT was used for qualitative and mixed‐methods studies and evaluates methodological quality across five domains including the appropriateness of the research questions, adequacy of data collection methods, integration of qualitative and quantitative findings, interpretation of results and coherence between data sources and conclusions (Hong et al. 2018). MMAT items are similarly rated using categorical responses (‘Yes’, ‘No’ or ‘Can't tell’). These tools were selected because they are widely used and suitable for appraising diverse study designs within mixed‐methods systematic reviews.

Quality appraisal scores for all included studies are presented in Table 1. Item‐level quality appraisal results for all included studies are presented in Files S2 and S3.

**TABLE 1:** Summary of included studies.

Authors (date), country	Study design and population	Variables and measurement tools	Other mental illnesses/non‐ASD comparators	Key findings	Quality assessment tool and score
Adams et al. (2021), South Africa	Design: Cross‐sectional questionnaire Sample size: *n* = 40 Diagnoses: ASD (diagnosed by school) Gender: 82% male Age: 3–9.6 years Ethnicity: Unreported	Food insecurity: Unreported Feeding behaviours: Behavioural Paediatric Feeding Assessment Scale (BPFAS)	None	Prevalence of feeding behaviours: Most common food groups consumed: starches (95.71%), snacks (92.10%) and dairy (88.82%). Least common food groups: meat/fish (80.70%), fruits (73.02%) and vegetables (70.05%) Older children ate more food varieties (*F* = 351.2, *p* = 0.00, *R* ^2^ = 0.905) Relationship between ASD severity and number of foods eaten (−0.353) (*p* = 0.048)	Tool: AXIS Score: 18/20 Percentage: 90%
Al‐Kindi et al. (2020), Oman	Design: Cross‐sectional questionnaire Sample size: *n* = 375 (*n* = 163 ASD, *n* = 212 TD) Diagnoses: ASD (diagnosed by DSM‐5 criteria) Gender: 233 males, 142 females Age: 4–13 years Ethnicity: Unreported	Food insecurity: Unreported Feeding behaviours: Food Frequency Questionnaire (FFQ)	Non‐ASD	Prevalence of feeding behaviours: The average number of items within every food group was significantly higher for TD children as compared to children with ASD(*p* < 0.001) Less than 50% of the vegetable (47%) and fast food (36%) items were eaten by children with ASD, whereas the TD children consumed more than 50% from every food group (63%–86%) Of the 11 types of bread listed, only 6 appeared to be the most popular among children The desserts appeared to be the most popular in the food groups, with 9 out of the 14 listed items being consumed by more than 50% of the children with ASD	Tool: AXIS Score: 16/20 Percentage: 80%
Alharbi (2024), Saudi Arabia	Design: Cross‐sectional survey Sample size: *n* = 150 Diagnoses: ASD (self‐reported) Gender: Female 66% Age: 21–30 50%, 31–40 40.7%, 41–50 9.3% Ethnicity: Unreported	Food insecurity: Unreported Feeding behaviours: Adapted Checklist for Autism Spectrum Disorder (CASD)	None	Prevalence of feeding behaviours: Changes in the eating behaviour of children with ASD were found to differ significantly (*p* < 0.05) based on the number of children with ASD, the age of the children with ASD, the gender of the children with ASD, and the severity of their ASD symptoms Changes to dinner‐time routines were found to differ significantly (*p* < 0.05) based on the age of the children with ASD. Also, changes to morning routines were found to differ significantly (*p* < 0.05) based on the age of the children with ASD, their gender, and the severity of their ASD symptoms Impact of COVID‐19 to the family had a significant impact to eating behaviour and daily routines of the children with ASD	Tool: AXIS Score: 16/20 Percentage: 80%
Amin et al. (2022), Pakistan	Design: Cross‐sectional questionnaire Sample size: *n* = 180 (*n* = 60 ASD, *n* = 120 TD) Diagnoses: ASD (diagnosed by school). Gender: 60% male Age: 5–12 years Ethnicity: Unreported	Food insecurity: Unreported Feeding behaviours: Food Preference Questionnaire (FPQ) & Brief assessment of mealtime behaviour inventory (BAMBI)	Non‐ASD	Prevalence of feeding behaviours: Limited variety was exhibited often and at almost every meal by 31.7% of the participants with ASD, and by 19.2% of the TD participants Food refusal was exhibited often or at almost every meal by 30% of the participants with ASD, and by only 6.7% of the TD participants Snacks were the most preferred, and vegetables were the least preferred food group both for children with and without ASD Higher BMI for age percentile was found to be associated with greater preference for food items in the vegetable group, for children with ASD (*r* = 0.327, *N* = 60, *p* = 0.011)	Tool: AXIS Score: 18/20 Percentage: 90%
Anderson et al. (2023), USA	Design: Cross‐sectional data analysis Sample size: *n* = 12 143 Diagnoses: ASD (self‐reported) Gender: Unreported Age: 3–17 years Ethnicity: White (39.5%); Black (20.9%), other (7.9%) and Hispanic (31.7%)	Food insecurity: National Survey of Children's Health (NSCH) Feeding behaviours: Unreported	Other special care needs and non‐ASD	Prevalence of Food Insecurity: Children with ASD experienced significantly greater levels of general hardship than those of children with and without other SHCN (40.0% vs. 28.9% and 19.6%, respectively); however, they were not significantly more likely to receive cash assistance than either comparison group Children with ASD were also more likely than children with no SHCN to be food insecure (14.0% vs. 7.9%) despite having higher rates of SNAP participation (54.0% vs. 44.0%)	Tool: AXIS Score: 20/20 Percentage: 100%
Aponte and Romanczyk (2016), USA	Design: Cross‐sectional questionnaire Sample size: *n* = 38 Diagnoses: ASD (self‐reported) Gender: 32 males, 6 females Age: 3–12 years Ethnicity: Unreported	Food insecurity: Unreported Feeding behaviours: FFQ & BAMBI	None	Prevalence of feeding behaviours: Post hoc analysis using the Bonferroni method indicated that children were more likely to consume dairy items at least daily, and parents were less likely to rate dairy items as ‘Never’ consumed at all compared to vegetables, proteins, fruits, starches, and combination foods. Protein food items were most likely to be rated as ‘Never’ consumed Autism severity predicted scores on a measure of feeding problems (*r* = 0.45, *p* = 0.001), as well as the duration of negative vocalisations during meal observations. Family environment alone did not explain feeding difficulties	Tool: AXIS Score: 17/20 Percentage: 85%
Attlee et al. (2015), UAE	Design: Cross‐sectional questionnaire Sample size: *n* = 23 Diagnosis: ASD (diagnosed by DSM‐5 criteria) Gender: 18 male, 5 female Age: 5–16 years Ethnicity: Unreported	Food insecurity: unreported Feeding behaviours: BAMBI	None	Prevalence of feeding behaviours: Most preferred foods were starches, whereas protein (meat, fish, poultry, beans and legumes) was least preferred Mean food refusal by food group: Combined food groups: 59.1% (SD: 20.6) Fruit: 63.3% (SD: 24.1) Protein: 67.4% (SD: 18.7) Starch: 44.2% (SD: 22.0) Vegetables: 59.0% (SD: 28.8) Dairy: 52.1% (SD: 26.9) Average rejection of around 40% of total foods, mentioned as ‘never consumed’ Around 70% of children ‘occasionally’ liked to try new foods	Tool: AXIS Score: 16/20 Percentage: 80%
Ausderau and Juarez (2013), USA	Design: Interviews Sample size: *n* = 6 Diagnoses: ASD (diagnosed by physician) Gender: Unreported Age: 2–7 years Ethnicity: White (*n* = 5) and Hispanic (*n* = 1)	Food insecurity: Unreported Feeding behaviours: Interviews	None	Prevalence of feeding behaviours: Mothers expressed their child with ASD often refused to sit at the family table or behaviours disrupted other family members Mothers identified strategies such as implementing ideas from interventionists or ones stumbled upon (i.e., playing with food), encouraging the child to explore novel foods in a stepwise manner (i.e., simply smelling the food or tolerating it on their plate), abiding by the child's ‘unique rules’ for food preparation and presentation and using a variety of methods to keep the child at the table These women often crafted a minimum of 2 menus, preparing one meal for the family and at least one special meal for their child with ASD attempting to abide by their food selectivity and following a specific routine with food preferences and presentation. These mothers often had a ‘backup’ meal or food in case the child with ASD refused their prepared food or was having a difficult day	Tool: MMAT Score: 7/7 Percentage: 100%
Bandini et al. (2017), USA	Design: Cross‐sectional questionnaire Sample size: *n* = 18 Diagnoses: ASD (diagnosed by parents using ADI‐R) Gender: 89% male Age: Mean 6.8 Ethnicity: Unreported	Food insecurity: Unreported Feeding behaviours: FFQ & Meals in Our Household Questionnaire (MIOH)	None	Prevalence of feeding behaviours: The children's food refusal improved between baseline and follow‐up. The overall percentage of foods refused of those offered decreased from 47% at baseline to 31% at follow‐up (*p* = 0.005) Fruit and vegetable refusal showed similar decreases between baseline and follow‐up. The absolute number of foods refused also declined; participants refused an average of 51 foods at baseline and 33 foods at follow‐up (*p* = 0.01) Almost two‐thirds of participants (65%) had a decrease in food repertoire between baseline and follow‐up, whereas 29% (*n* = 5) increased their food repertoire. Consumption of fruits and vegetables increased about one serving per day between the two time points (2.2 vs. 3.4 servings per day, *p* = 0.06)	Tool: MMAT Score: 6/7 Percentage: 86%
Brzóska et al. (2021), Poland	Design: Cross‐sectional questionnaire Sample size: *n* = 75 (*n* = 41 ASD, *n* = 34 non‐ASD) Diagnoses: ASD (diagnosed by psychiatrist) Gender: 72.4% male Age: 2–12 years Ethnicity: Caucasian (100%)	Food insecurity: Unreported Feeding behaviours: Self‐created questionnaire	Non‐ASD	Prevalence of feeding behaviours: Children with ASD, as compared to the healthy peers, presented a shortened time of breastfeeding (the children fell asleep at the breast) (*p* = 0.04), a delayed introduction of dairy products (*p* = 0.001), the need of more trials to introduce new foods (*p* = 0.006), a delayed introduction of foods with solid and lumpy structure (*p* = 0.004), a longer duration of bottle feeding (*p* = 0.005), delayed attempts to eating using own hands (*p* = 0.006) and needed a greater support of parents to divert their attention from food during eating (*p* = 0.05)	Tool: AXIS Score: 18/20 Percentage: 90%
Brochier et al. (2023), USA	Design: Cross‐sectional data analysis Sample size: *n* = 1321 Diagnoses: ASD (self‐reported) Gender: 77.9% male Age: 0–17 years Ethnicity: White (43.1%), Hispanic (34%), Black (14.4%), other (4.8%) and Asian (3.7%)	Food insecurity: NSCH Feeding behaviours: Unreported	None	Prevalence of food insecurity: Food insecurity was significantly associated with higher disease prevalence and severity for ASD (aOR: 2.1; 95% CI: 1.5–2.7) Caregiver underemployment, low social support, and discrimination were significantly associated with higher disease prevalence of ASD For each additional social risk factor a child was exposed to, their odds of having each condition increased: ASD (aOR: 1.4, 95% CI: 1.3, 1.5)	Tool: AXIS Score: 20/20 Percentage: 100%
Canals‐Sans et al. (2021), Spain	Design: Cross‐sectional questionnaire Sample size: *n* = 451 (*n* = 117 ASD; *n* = 333 TD) Diagnoses: ASD (diagnosed by DSM‐5 criteria) Gender: 83% male Age: 3–12 years Ethnicity: Autochthon (80%)	Food insecurity: Unreported Feeding behaviours: FFQ	Non‐ASD	Prevalence of feeding behaviours: Compared to children with TD, pre‐schoolers with ASD consumed fewer raw vegetables and less fish and eggs, whereas primary school children consumed fewer legumes, raw vegetables, citrus fruits, cheese/yogurt and olive oil and more meat All groups consumed an excess of sugar but those with ASD consumed even a greater amount than their peers	Tool: AXIS Score: 19/20 Percentage: 95%
Carter Leno et al. (2022), England	Design: Cross‐sectional data analysis Sample size: *n* = 8982 Diagnoses: ASD (using SCDC) Gender: 51% male Age: 7–14 years Ethnicity: Unreported	Food insecurity: Unreported Feeding behaviours: Food Frequency Questionnaire	None	Prevalence of feeding behaviours: Analyses found a small indirect pathway from autistic traits intercept to eating disorder behaviours via fussy eating slope (*b* = 0.017, 95% CI = 0.002–0.032, *p* = 0.026), with higher levels of autistic traits at age 7 years being associated with a shallower decline in fussy eating, which in turn was associated with greater eating disorder behaviours	Tool: MMAT Score: 7/7 Percentage: 100%
Chen et al. (2024), China	Design: Cross‐sectional survey Sample size: *n* = 509 Diagnoses: ASD (using SRS‐2) Gender: Female 53% Age: 3.5 Ethnicity: Unreported	Food insecurity: Unreported Feeding behaviours: CPEBQ; SSP2‐2	None	Prevalence of feeding behaviours: Observed a positive association between autistic traits and eating behaviour problems among typically developed children. Sensory processing patterns significantly mediated the impact of autistic traits on children's eating‐related behaviours and home nurturing environment also moderated this relationship	Tool: AXIS Score: 15/20 Percentage: 75%
Chistol et al. (2018), USA	Design: Cross‐sectional questionnaire Sample size: *n* = 111 (*n* = 53 ASD; *n* = 58 TD) Diagnoses: ASD (diagnosed using ADI‐R) Gender: 83% male Age: 3–11 years Ethnicity: 83% White	Food insecurity: Unreported Feeding behaviours: FFQ & Sensory Profile (SP)	Non‐ASD	Prevalence of feeding behaviours: Compared to the TD group, children with ASD had significantly higher levels of food refusal, significantly lower levels of food repertoire, and less variety of fruits and vegetables On average, children with ASD scored lower on the Sensory Profile than TD children, indicating more atypical sensory processing Children with ASD who scored in the Atypical versus Typical range for Oral Sensory Sensitivity had significantly higher levels of food refusal (48.2% vs. 33.2%, respectively, *p* = 0.01). Compared to children with ASD who scored in the Typical range for Oral Sensory Over‐sensitivity, those who scored in the Atypical range had twice the level of food refusal (52% vs. 25%, *p* < 0.001). Fruit variety was significantly lower for children who scored in the Atypical versus Typical range for Oral Sensory Over‐sensitivity (5.3 vs. 8.2, *p* = 0.003) but was not significantly different for those who scored in the Atypical versus Typical range on the Oral Sensory Sensitivity measure.	Tool: AXIS Score: 17/20 Percentage: 85%
Cihon et al. (2020), USA	Design: Intervention Sample size: *n* = 3 Diagnoses: ASD (diagnosed using SSiS) Gender: 2 boys, 1 girl Age: 5–11 years Ethnicity: Unreported	Food insecurity: Unreported Feeding behaviours: Intervention	None	Prevalence of feeding behaviours: The results of a reversal design demonstrated that selection of the low‐preferred food only occurred following the introduction of the intervention, and all three participants engaged in flexible responding as a result of the intervention	Tool: MMAT Score: 6/7 Percentage: 86%
Conine and Vollmer (2019), USA	Design: Observation Sample size: *n* = 26 Diagnoses: ASD (unreported) Gender: 22 males, 4 females Age: 2–12 years Ethnicity: Unreported	Food insecurity: Unreported Feeding behaviours: Reinforcer Assessment for Individuals with Severe Disabilities (RAISD)	None	Prevalence of feeding behaviours: Results indicated that edible items were more likely than leisure items to rank highly in our preference assessments, in concurrence with prior research. However, leisure items were also selected more often overall than in prior research, and significant individual variation was observed	Tool: MMAT Score: 6/7 Percentage: 86%
Crasta et al. (2014), India	Design: Cross‐sectional questionnaire Sample size: *n* = 97 (*n* = 41 ASD; *n* = 56 ID) Diagnoses: ASD (diagnosed using ICD‐10) Gender: 72 boys, 25 girls Age: 3–10 years Ethnicity: Unreported	Food insecurity: Unreported Feeding behaviours: BAMBI & SP	Intellectual disabilities	Prevalence of feeding behaviours: The prevalence of FP were 61% and 46.4% among children with autism and ID, respectively Feeding problems were severe among children with ASD (*p*: 0.001), especially in young children with ASD (*p*: 0.05), and gender was not related to FP Disruptive meal‐time behaviours (*p*: 0.001) and food over‐selectivity (*p*: 0.02) were significantly more among children with ASD in the bivariate and multivariate analysis Feeding problems and various dimensions of sensory processing were significantly associated after controlling the confounders	Tool: AXIS Score: 15/20 Percentage: 75%
Crippa et al. (2022), Italy	Design: Cross‐sectional questionnaire Sample size: *n* = 99 Diagnoses: ASD (diagnosed by DSM‐5 criteria) Gender: 84 boys, 15 girls Age: 1–5 years Ethnicity: Caucasian (89%), African (2%), Asian (2%), Hispanic (2%) and Bi‐racial (1%)	Food insecurity: Unreported Feeding behaviours: Children's Eating Behaviour Inventory (CEBI) & FFQ	None	Prevalence of feeding behaviours: Children with feeding problems showed more severe internalising symptoms and were more reactive to sensory stimuli than children without feeding problems We also found a significant relationship between higher levels of autistic features and greater feeding behaviour problems	Tool: AXIS Score: 19/20 Percentage: 95%
Crowley et al. (2020), USA	Design: Intervention Sample size: *n* = 7 Diagnoses: ASD (diagnosed by DSM‐5 criteria) Gender: 5 males, 2 females Age: 2–8 years Ethnicity: Unreported	Food insecurity: Unreported Feeding behaviours: Intervention	None	Prevalence of feeding behaviours: Alternative‐food consumption increased for 2 participants during asymmetrical choice when the feeder provided a preferred item for consuming the alternative food and no programmed consequence for consuming the change resistant food Alternative‐food consumption increased for the other 5 participants after the feeder exposed at least 1 food to single choice in which the feeder guided the participant to put the bite of alternative food in his or her mouth if he or she did not do so within 8 s of presentation	Tool: MMAT Score: 6/7 Percentage: 86%
Curtin et al. (2015), USA	Design: Cross‐sectional questionnaire Sample size: *n* = 111 (*n* = 53 ASD; *n* = 58 TD) Diagnoses: ASD (diagnosed using ADI‐R) Gender: 80.5% male Age: 3–11 years Ethnicity: 86% White	Food insecurity: Unreported Feeding behaviours: Youth/Adolescent Food Frequency Questionnaire (YAQ) & FFQ	Non‐ASD	Prevalence of feeding behaviours: Compared to TD children, children with ASD were more likely to have high food selectivity (*β* = 5.44 in ASD, *p* = 0.003; *β* = 3.45 in TD, *p* = 0.04), and their parents reported more mealtime behaviour problems (mean of frequency scale scores 19.7 vs. 12.0, *p* < 0.01), higher spousal stress (*n* = 49 and 51, respectively; mean of spousal stress scale scores 10.7 vs. 6.9, *p* < 0.01), and influence on what other family members ate (mean influence scale scores 6.2 vs. 4.2, *p* < 0.01) High food selectivity was associated with mealtime behaviour problems in both groups	Tool: AXIS Score: 15/20 Percentage: 75%
Curtiss and Ebata (2019), USA	Design: Interviews Sample size: *n* = 16 Diagnoses: ASD (recruited through autism charities) Gender: Unreported Age: 5–12 years Ethnicity: White (*n* = 10), Black (*n* = 1), Latino (*n* = 1), Asian (*n* = 1) and multi‐ethnic (*n* = 3)	Food insecurity: Unreported Feeding behaviours: Interviews	None	Prevalence of feeding behaviours: Mealtimes are a stressful task for families of children with ASD, but they are also joyous, important, satisfying, and rich with potential for feeling connected and supported Doing other activities helped (alongside mealtimes) such as homework Parents were worried that their children did not eat enough, did not eat a varied enough diet, and did not want to eat at the times the parent thought they should eat	Tool: MMAT Score: 7/7 Percentage: 100%
Curtiss and Ebata (2021), USA	Design: Interviews Sample size: *n* = 16 Diagnoses: ASD (recruited through autism charities) Gender: Unreported Age: 5–12 years Ethnicity: White (*n* = 10), Black (*n* = 1), Latino (*n* = 1), Asian (*n* = 1), multi‐ethnic (*n* = 3)	Food insecurity: Unreported Feeding behaviours: Interviews	None	Prevalence of feeding behaviours: An understanding of autism and mealtimes should be re‐centered on the family rather than the child with ASD	Tool: MMAT Score: 7/7 Percentage: 100%
Demchuk et al. (2024), USA	Design: Case study Sample size: *n* = 1 Diagnoses: ASD (previous diagnosis) Gender: Male 100% Age: 5 Ethnicity: Hispanic	Food insecurity: Unreported Feeding behaviours: Case study	None	Prevalence of feeding behaviours: Synchronous reinforcement (SSR) was an effective intervention for increasing self‐feeding, as well as methods for fading SSR as a treatment component. We discuss the potential for SSR as a trauma‐informed approach, and the importance of caregiver collaboration and programming for generalisation	Tool: MMAT Score: 6/7 Percentage: 86%
Demir and Özcan (2021), Turkey	Design: Cross‐sectional questionnaire Sample size: *n* = 204 (*n* = 104 ASD, *n* = 100 TD) Diagnoses: ASD (diagnosed by DSM‐5 criteria) Gender: 80.8% male Age: 3–12 years Ethnicity: Unreported	Food insecurity: Unreported Feeding behaviours: Child Eating Behaviour Questionnaire (CEBQ) & Parental Feeding Style Questionnaire (PFSQ)	Non‐ASD	Prevalence of feeding behaviours: Children with ASD were difficult to feed as babies, experienced more problems in the transition to supplementary food, were more selective about food, and were fed diets with a more limited variety than the control group The BMI *z*‐scores for children with ASD were higher than those for children without ASD, whereas their height *z*‐scores were lower Children with ASD displayed more responsiveness to food, emotional overeating, enjoyment of food, desire for drinks, emotional undereating, and food selectivity behaviours, whereas the parents of these children were found to use more emotional feeding, instrumental feeding, and tolerance‐controlled feeding styles than the parents of the controls	Tool: AXIS Score: 15/20 Percentage: 75%
Dhillon‐Burrows et al. (2023), England	Design: Interviews Sample size: *n* = 15 Diagnoses: ASD (self‐reported) Gender: 8 males, 6 females Age: 6–16 years Ethnicity: White British (*n* = 13), White Irish (*n* = 1) and White and Black Caribbean (*n* = 1)	Food insecurity: Interviews Feeding behaviours: Interviews	None	Prevalence of feeding behaviours/food insecurity: Eating behaviours in children with ASD were both positively and negatively affected by the COVID‐19 lockdown Whilst all parents felt under pressure with extra care responsibilities, some described less concern over food intake during the first lockdown because they were able to support their child's mealtimes and have new food experiences By the end of the lockdowns, many parents reported worsening of eating behaviours, with children more likely to have a restricted diet, and eating more snack foods. Food shortages also triggered additional stress with parents unable to access their child's preferred food	Tool: MMAT Score: 7/7 Percentage: 100%
Diolordi et al. (2014), Italy	Design: Cross‐sectional questionnaire Sample size: *n* = 68 (*n* = 33 ASD; *n* = 35 TD) Diagnoses: ASD (diagnosed by DSM‐5 criteria) Gender: 81.8% male Age: Mean 58.3 months Ethnicity: Unreported	Food insecurity: Unreported Feeding behaviours: Food frequency & CEBI	Non‐ASD	Prevalence of feeding behaviours: A statistically significant difference was observed between the two groups for the consumption of milk, yogurt, pulses, rice and fruit juices (*p* B 0.005) We observed a significant difference in the analysis of CEBI results when considering the 6‐ to 9.5‐year‐aged subgroup with autism vs. control subgroup (103.50 and 110.14, respectively)	Tool: AXIS Score: 15/20 Percentage: 75%
Dovey et al. (2019), England	Design: Cross‐sectional questionnaire Sample size: *n* = 486 (ARFID *n* = 29, ASD *n* = 56, PE *n* = 143) or no reported difficulties (*n* = 259) Diagnoses: ASD (self‐reported) Gender: 41 males, 14 females Age: Mean 95.8 months Ethnicity: European decent (90.2%), mixed heritage (6.8%) and other (3%)	Food insecurity: Unreported Feeding behaviours: CEBQ & The Sensory Experiences Questionnaire (SEQ)	Non‐ASD	Prevalence of feeding behaviours: The ARFID, ASD and PE groups had eating difficulties (all *p* < 0.001), behavioural problems and sensory hypersensitivity, relative to the typically developing group, and differed significantly on only some of the dimensions assessed Specifically, the ARFID group had the lowest food‐responsiveness and differed significantly from the PE and typically developing (but not from ASD) groups while the ASD group had significantly greater behavioural problems and social and non‐social sensitivity than all other groups	Tool: AXIS Score: 15/20 Percentage: 75%
Farag et al. (2022), England	Design: Observations Sample size: *n* = 536 (*n* = 247 ASD) Diagnoses: ASD (recruited by local neurodevelopmental team) Gender: 401 males, 135 females Age: 0–20 years Ethnicity: Unreported	Food insecurity: Unreported Feeding behaviours: Observation	Non‐ASD	Prevalence of feeding behaviours: ARFID is more prevalent among younger patients (4–9 years) (OR 0.93; 95% CI: 0.88–0.98; *p* = 0.006) and in children with comorbid ASD (OR 2.04; 95% CI: 1.43–2.92; *p* < 0.001). Younger age, comorbid ASD, and male sex (OR 2.01; 95% CI: 1.33–3.04; *p* = 0.001) significantly predicted ARFID Diet range and male sex significantly predicted nutritional inadequacy, whereas comorbid ASD did not. A trend was seen between younger age and nutritional inadequacy	Tool: AXIS Score: 13/20 Percentage: 65%
Fernandez et al. (2024), Philippines	Design: Cross‐sectional survey Sample size: *n* = 115 Diagnoses: ASD (DSM‐5 criteria) Gender: Female 93% Age: 31.9 mean Ethnicity: Unreported	Food insecurity: Unreported Feeding behaviours: Adaption of the Mealtime Survey by Provost et al.	None	Prevalence of feeding behaviours: All of the 115 study subjects reported at least one problematic feeding behaviour, with picky eating being the most frequent (61.74%) Significantly, more feeding difficulties were observed among the children with reported early feeding difficulties during their 2nd and 3rd year of life There were no documented statistically significant changes in feeding behaviours during the past six months of the COVID‐19 pandemic	Tool: AXIS Score: 16/20 Percentage: 80%
Graf‐Myles et al. (2013), USA	Design: Cross‐sectional data analysis Sample size: *n* = 120 (*n* = 69 ASD; *n* = 14 developmental delay; *n* = 37 TD) Diagnoses: ASD (diagnosed by DSM‐5 criteria) Gender: Unreported Age: 1–6 years Ethnicity: Unreported	Food insecurity: Unreported Feeding behaviours: Short Sensory Profile (SSP)	Non‐ASD	Prevalence of feeding behaviours: Children with ASD did not differ significantly from children with other developmental delays on any dietary measures. Although there were differences in average intake of some nutrients between autism and typical controls, only calcium and dairy were also less likely to be consumed in adequate amounts by the autism group Children in the autism group not following a restricted diet received significantly worse Healthy Eating Index‐2005 scores than those following a restricted diet and typical controls. These differences were not nutritionally significant	Tool: AXIS Score: 19/20 Percentage: 95%
Gray et al. (2022), USA	Design: Focus groups Sample size: *n* = 41 Diagnoses: ASD (clinically diagnosed) Gender: 73% male Age: 2–17 years Ethnicity: White (39%), Hispanic/Latino (34%) and other (27%)	Food insecurity: Unreported Feeding behaviours: Food reporting booklet	None	Prevalence of feeding behaviours: Children with ASD were significantly less likely to meet the recommendation for calcium compared with children in the general population (66.7% vs. 53.1%, odds ratio [OR] = 3.1; *p* = 0.002). Riboflavin and vitamin B12 intakes were significantly lower among children with ASD (*p* < 0.001) Focus group results indicated parental concerns on children's diet and mealtime behaviours. Parents discussed the quality of existing nutrition information to be a concern, reported wanting intervention content on effective feeding strategies and healthy eating specific to children with ASD, and wanted to receive a nutrition intervention in multiple delivery formats	Tool: AXIS Score: 15/20 Percentage: 75%
Guller and Yaylaci (2024), Turkey	Design: Cross‐sectional questionnaire Sample size: *n* = 156 Diagnoses: ASD (diagnosed by CARS) Gender: Male 87% Age: Mean 8.08 Ethnicity: Unreported	Food insecurity: Unreported Feeding behaviours: BAMBI	None	Prevalence of feeding behaviours: The mean score of CARS was 41.25 ± 6.16, the mean score of BAMBI was 45.67 ± 11.87, and the mean score of CSHQ was 51.67 ± 10.17 There was a positive correlation between CARS and BAMBI, CSHQ, HAD‐A and HAD‐D subscales. There was a positive correlation between BAMBI and CHSQ, HAD‐A and HAD‐D subscales. There was a positive correlation between the CSHQ, HAD‐A and HAD‐D subscales There was a negative correlation between the age of the child with ASD, food refusal and resistance to bedtime	Tool: AXIS Score: 15/20 Percentage: 75%
Hladik et al. (2025), USA	Design: Interviews Sample size: *n* = 13 Diagnoses: ASD (self reported) Gender: Female 100% Age: Unreported Ethnicity: Unreported	Food insecurity: Unreported Feeding behaviours: Interview	None	Prevalence of feeding behaviours: Four major themes were identified: In‐Home Intervention, Parent Skill and Knowledge, Increased Social Participation and Parent Responsibilities and Challenges Sub‐themes provided descriptions of learning practical tools to support their child, increasing self‐efficacy, and impacts on family life. Mothers described an increase in tangible skills that were easily practiced in the home environment that improved their confidence and self‐efficacy in feeding their children They also described how participation did require more work and time commitment for them beyond their regular responsibilities	Tool: MMAT Score: 7/7 Percentage: 100%
Hubbard et al. (2014), USA	Design: Cross‐sectional questionnaire Sample size: *n* = 111 (*n* = 53 ASD; *n* = 58 TD) Diagnoses: ASD (diagnosed using ADI‐R) Gender: 80.5% male Age: 3–11 years Ethnicity: 79.5% White	Food insecurity: Unreported Feeding behaviours: FFQ	Non‐ASD	Prevalence of feeding behaviours: Children with ASD were significantly more likely to refuse foods based on texture/consistency (77.4% vs. 36.2%), taste/smell (49.1% vs. 5.2%), mixtures (45.3% vs. 25.9%), brand (15.1% vs. 1.7%) and shape (11.3% vs. 1.7%) No differences between groups were found for food refusal based on temperature, foods touching other foods or colour	Tool: AXIS Score: 18/20 Percentage: 90%
Huxham et al. (2019), England	Design: Cross‐sectional questionnaire Sample size: *n* = 325 Diagnosis: ASD (self‐reported), Asperger syndrome and other Gender: 85% male Age: 3–16 years Ethnicity: unreported	Food insecurity: Unreported Feeding behaviours: Self‐created questionnaire	None	Prevalence of feeding behaviours: Differences in food preferences (*p* < 0.05) for all foods apart from unrefined carbohydrates (*p* = 0.021) and vegetables (*p* = 0.855) (Chi square statistics unreported) Food preferences by food group: Fruit: always refused (19.2%) vs. will eat readily (41.6%). Fruit juice: always refused (20.5%) vs. will eat readily (45.7%). Vegetables: always refused (25.5%) vs. will eat readily (26.9%). Starchy vegetables: always refused (14.4%) vs. will eat readily (41.6%). Unrefined carbohydrate: always refused (27.6%) vs. will eat readily (31.1%). Refined carbohydrate: always refused (4.8%) vs. will eat readily (64.4%). Eggs: always refused (44.3%) vs. will eat readily (23.3%). Unprocessed meat: always refused (27.5%) vs. will eat readily (35.9%). Processed meat: always refused (16.1%) vs. will eat readily (52.4%). Meat alternatives: always refused (57.1%) vs. will eat readily (11.3%). Dairy: always refused (14.8%) vs. will eat readily (50.7%) The majority of children (64.7%, *n* = 191) were influenced by food presentation including specific brands and packaging The majority of children (75.6%, *n* = 223) would repeatedly choose the same foods from a limited range during meals Refined carbohydrates were the most popular food group (white bread, cake, biscuits, pastries, and refined cereals) where 64.4% (*n* = 186) of children would readily eat these (*p* < 0.001)	Tool: AXIS Score: 18/20 Percentage: 90%
Islam et al. (2024), Bangladesh	Design: Cross‐sectional questionnaire Sample size: *n* = 344 Diagnoses: ASD (self reported) Gender: Female 29.7% Age: 7.9 mean Ethnicity: Unreported	Food insecurity: Unreported Feeding behaviours: FFQ	Non‐ASD	Prevalence of feeding behaviours: Children with ASD were more likely to be overweight and obese compared to the non‐ASD group (RRR: 2.85, 95% CI 1.28–6.34, *p*‐value 0.011). ASD children had lower dietary diversity than non‐ASD children (RRR: 18.57, 95% CI 4.49–76.77, *p*‐value < 0.001) Children with ASD had a significantly lower daily frequency of intake of food from starchy roots, sugars, preserves and syrups, meat, fish and eggs, milk and milk products than non‐ASD children (*p*‐value < 0.05). However, consumption of cereals, vegetables and fruits, fats and oils, and beverages was almost similar for both groups of children	Tool: AXIS Score: 16/20 Percentage: 80%
İslamoğlu et al. (2024), Turkey	Design: Cross‐sectional observation Sample size: *n* = 109 Diagnoses: ASD (self reported) Gender: Male 66.1% Age: 3–18 years Ethnicity: Unreported	Food insecurity: Unreported Feeding behaviours: 24‐h dietary recall	None	Prevalence of feeding behaviours: The rate of food selectivity was found to be 59.6%; 28.4% of the participants received additional support during mealtimes, 13.8% followed various special diets including gluten‐free and casein‐free diets. Most of the participants' energy intake (60.6%), vitamin D (100.0%) and calcium (71.6%) were below reference values. The majority of the participants' vitamin A (92.7%) and sodium (92.7%) intakes were above the recommended values	Tool: AXIS Score: 16/20 Percentage: 80%
Ismail et al. (2020), Malaysia	Design: Focus groups Sample size: *n* = 20 Diagnoses: ASD (recruited from specialist hospitals) Gender: Unreported Age: 4–7 years Ethnicity: Unreported	Food insecurity: Unreported Feeding behaviours: Focus groups	None	Prevalence of feeding behaviours: Inadequate knowledge was significantly correlated with low nutritional status of their children (*p* < 0.05) Three out of fourteen parents have reported that their children bear oral health issues exemplified by dental associated damage and inability to chew caused by feeding behaviour They further iterated this was due to the fact of their children's habit and preferences towards hard and crunchy textured foods	Tool: MMAT Score: 7/7 Percentage: 100%
Johnson et al. (2014), USA	Design: Cross‐sectional questionnaire Sample size: *n* = 256 Diagnoses: ASD (diagnosed by DSM‐5 criteria) Gender: 84% male Age: 2–11 years Ethnicity: 90% Caucasian	Food insecurity: Unreported Feeding behaviours: BAMBI & SSP	None	Prevalence of feeding behaviours: For each unit increase in the RBRS total score, the BAMBI total score increased by 0.28 [*t* (249) = 10.55, *p*\0.0001]. Similarly, for each unit increase in the SSP total score, the BAMBI total score decreased by 0.26 [*t* (216) = −8.64, *p*\0.0001] Controlling for medications, for each unit increase of the BAMBI total scores, the HEI decreased by [0.31 (*t* (211) = −4.21), *p*\0.0001] indicating that nutrition quality decreased as problem feeding behaviours increased	Tool: AXIS Score: 15/20 Percentage: 75%
Kang et al. (2022), Singapore	Design: Cross‐sectional questionnaire Sample size: *n* = 67 Diagnoses: ASD (diagnosed by DSM‐5 criteria) Gender: 89% male Age: 1–7 years Ethnicity: Chinese (68.7%), Malay (22.4%), Indian (4.5%) and other (4.5%)	Food insecurity: Unreported Feeding behaviours: BAMBI, BPFAS, & Caregiver Feeding Style Questionnaire (CFSQ)	None	Prevalence of feeding behaviours: Of the 67 children, 28.4% had feeding difficulties (high BPFAS total frequency score) Caregiver feeding styles were authoritarian (34.8%) or indulgent (39.4%). Child characteristics did not significantly predict for the severity of feeding difficulties. Univariate analysis revealed that authoritarian feeding style (*p* Z 0.001) and ABC hyperactivity score (*p* Z 0.006) were significantly associated with BPFAS severity score	Tool: AXIS Score: 15/20 Percentage: 75%
Karpur et al. (2022), USA	Design: Cross‐sectional data analysis Sample size: *n* = 1515 Diagnoses: ASD (self‐reported) Gender: Unreported Age: Unreported Ethnicity: Unreported	Food insecurity: Autism Speaks' Food Insecurity Survey (AFIS) & Household Pulse Survey (HPS) Feeding behaviours: Unreported	Non‐ASD	Prevalence of food insecurity: After adjusting for background differences, households of children on the autism spectrum in the ASFIS were about four times more likely to be food insecure than households in the general population contained in the HPS data (OR ¼ 3.7; 95% CI: 3.1e4.4)	Tool: AXIS Score: 19/20 Percentage: 95%
Karpur et al. (2021), USA	Design: Cross‐sectional data analysis Sample size: *n* = 2238 Diagnoses: ASD (self‐reported) Gender: Unreported Age: 0–17 years Ethnicity: Unreported	Food insecurity: NSCH & National Survey of Children with Special Health Care Needs (NSCHCN) Feeding behaviours: Unreported	Intellectual disabilities	Prevalence of food insecurity: A substantially higher proportion of children with ASD + ID were food insecure (44%), followed by children with ASD only (40%), children with other disabilities (33%), and those with no disabilities (20%) Households of children with ASD + ID were about two times more likely to be food insecure than the households of children without disabilities. Further, the households of children with ASD were 1.5 times more likely, and those with other disabilities were 1.3 times more likely to be food insecure than the households of children without disabilities	Tool: AXIS Score: 18/20 Percentage: 90%
Kazek et al. (2021), Poland	Design: Cross‐sectional questionnaire Sample size: *n* = 75 (*n* = 41 ASD, *n* = 34 TD) Diagnoses: ASD (diagnosed using ADOS‐2) Gender: 68.3% male Age: 3–10 years Ethnicity: Unreported	Food insecurity: Unreported Feeding behaviours: Self‐created questionnaire	Non‐ASD	Prevalence of feeding behaviours: The children with ASD fuss during mealtimes more frequently, they require entertaining and diverting their attention, they are fed by parents, and they consume their meals away from the table. The significant difference found in the use of utensils and food selectivity works to the disadvantage of the study group	Tool: AXIS Score: 15/20 Percentage: 75%
Khatoon and Senapati (2024), India	Design: Intervention Sample size: *n* = 1 Diagnoses: ASD (previous diagnosis) Gender: Male Age: 3.3 Ethnicity: Indian	Food insecurity: Unreported Feeding behaviours: Sensory Profile & PediEAT	None	Prevalence of feeding behaviours: A combination of oro‐motor stimulation and aroma therapy is an effective intervention in decreasing oral sensory issues and thus improving feeding skills in children with ASD	Tool: MMAT Score: 7/7 Percentage: 100%
Kim and Kwon (2022), USA	Design: Cross‐sectional data analysis Sample size: *n* = 529 Diagnoses: ASD (self‐reported) Gender: 83.2% male Age: 10–17 years Ethnicity: Hispanic (38.34%), non‐Hispanic white (43.89%), non‐Hispanic black (11.90%), non‐Hispanic Asian (1.36%) and others (4.50%)	Food insecurity: Likert Scale Feeding behaviours: Likert Scale	None	Prevalence of feeding behaviours/food insecurity: Two‐parent households, less healthy parents and households, households with smokers, poor sleep quality, and greater participation in organised activities were associated with a higher likelihood of overweight in children with ASD (all *p* < 0.05) In terms of obesogenic factors, children who slept well had statistically significantly lower odds of being overweight than those who did not receive adequate sleep [OR = 0.38, 95% confidence interval (CI) = 0.19–0.71] According to parenting capacity, children from two‐parent households (OR = 5.70, 95% CI = 1.83–17.79) were more likely to be overweight than children from any other family structure (e.g., one parent, no parents, etc.) In terms of community and school activities, children who had participated in more organised activities after school or on weekends over the past 12 months were more likely to be overweight than those who had not (OR = 2.74, 95% CI = 1.35–5.55)	Tool: AXIS Score: 20/20 Percentage: 100%
Calisan Kinter et al. (2024), Turkey	Design: Cross‐sectional questionnaire Sample size: *n* = 111 Diagnoses: ASD (diagnosed by CARS) Gender: Male 73.9% Age: Mean 6.40 Ethnicity: Turkish 100%	Food insecurity: Unreported Feeding behaviours: CEBQ; SP	Non‐ASD	Prevalence of feeding behaviours: Children with ASD and ARFID had higher scores in CEBQ subscales relating to low appetite and lower scores on the subscales associated with weight gain Both groups of children with ASD scored lower than TDC on all PedsQL subscales and Children with ASD and ARFID had lower social QL scores than both groups SRS scores were highest in Children with ASD and ARFID, followed by children with ASD and TD children. CARS scores were similar in both groups of children with ASD, but higher than TDC Auditory, vision, touch, multi‐sensory, oral processing scores; as well as all quadrant scores, were significantly lower in children with ASD and ARFID. Oral sensory processing scores were found to be the most significant predictor of ARFID comorbidity in ASD and reliably predicted ARFID in children with ASD in the clinical setting	Tool: AXIS Score: 15/20 Percentage: 75%
Kral et al. (2023), USA	Design: RCT Sample size: *n* = 38 Diagnoses: ASD (diagnosed by DSM‐5 criteria) Gender: 94.7% male Age: 6–10 years Ethnicity: White (68%) Hispanic (13%)	Food insecurity: Unreported Feeding behaviours: Child Feeding Questionnaire (CFQ)	None	Prevalence of feeding behaviours: Children in the intervention group who consumed few FV at baseline and showed high engagement with the technology increased their FV intake by 1.5 servings/day (*p* < 0.01) Children's taste/smell sensitivity significantly predicted their FV intake (*p* = 0.0446); for each unit of lower taste/smell sensitivity (indicating greater sensory processing abnormalities), FV intake increased by 0.13 ± 0.1 servings/day	Tool: MMAT Score: 5/7 Percentage: 71%
Kral et al. (2015), USA	Design: Cross‐sectional questionnaire Sample size: *n* = 55 (*n* = 25 ASD) Diagnoses: ASD (confirmed by community provider) Gender: 72% male Age: 4–6 years Ethnicity: Non‐Hispanic (84%)	Food insecurity: Unreported Feeding behaviours: Child Food Neophobia Scale (CFNS), CFQ, PFSQ, SPCQ & CEBQ	Non‐ASD	Prevalence of feeding behaviours: Children with ASD with atypical oral sensory sensitivity exhibited greater food avoidance behaviours including reluctance to eat novel foods (*p* = 0.004), being selective about the range of foods they accept (*p* = 0.03), and undereating due to negative emotions (*p* = 0.02), than children with ASD with typical oral sensory sensitivity	Tool: AXIS Score: 17/20 Percentage: 85%
Lázaro and Pondé (2017), Brazil	Design: Interviews Sample size: *n* = 18 Diagnoses: ASD (confirmed by school) Gender: 100% male Age: 5–28 years Ethnicity: Unreported	Food insecurity: Unreported Feeding behaviours: Interviews	None	Prevalence of feeding behaviours: The most commonly consumed foods were rice, beans, potatoes, and blended soup or soup containing instant pasta. Mothers also mentioned a high consumption of various types of cookies, soft cheese‐filled buns, processed meats or hamburger, butter, soft drinks, pizza, popcorn, French fries, ice cream, chocolate, creamy yogurt, sausages and bacon. Some of the mothers mentioned that these preferences had been established through siblings or cousins who would consume these food items and offer them to the child, or otherwise, through children at school Some types of food were rejected by the children. Many children refused seasoning such as chives, parsley, tomatoes, peppers and coriander to be added to food. Vegetables and legumes were generally rejected. The majority of the children rejected fish. In addition, the majority refused to eat any kind of fruit or only specific kinds of fruit. Other children refused food without any juice or sauce such as barbecued meat	Tool: MMAT Score: 7/7 Percentage: 100%
Leader et al. (2021), Ireland	Design: Cross sectional questionnaire Sample size: *n* = 120 Diagnosis: ASD (diagnosed by DSM‐5 criteria) Gender: 77.5% male Age: 3–17 years Ethnicity: Unreported	Food Insecurity: unreported Feeding behaviours: Screening Tool of Feeding Problems for Children (STEP‐CHILD)	None	Prevalence of feeding behaviours: Overall frequency of feeding problems on the STEP‐CHILD was 90% (*n* = 108) Food refusal: 78.3% (*n* = 94), and food selectivity: 81.7% (*n* = 98) were the most common caregiver reported feeding problems Significant differences between those with (*M* = 96.75, SD = 23.53) and without (*M* = 114.42, SD = 23.53) food refusal, with respect to Total SSP; *t* (118) = 3.42, *p* < 0.05 Significant difference between those with (*M* = 97.70, SD = 24.52) and without (*M* = 113.36, SD = 19.27) food selectivity, with respect to Total SSP; *t* (118) = 2.8, *p* < 0.05	Tool: AXIS Score: 16/20 Percentage: 80%
Leiva‐García et al. (2019), Spain	Design: Cross‐sectional questionnaire Sample size: *n* = 146 (*n* = 55 ASD) Diagnoses: ASD (diagnosed by DSM‐5 criteria) Gender: 87% male Age: 6–18 years Ethnicity: Unreported	Food insecurity: Unreported Feeding behaviours: FFQ & Brief Assessment of Mealtime Behaviours in Children (BAMBIC)	Non‐ASD	Prevalence of feeding behaviours: The Chi squared test identified significant differences in food hyper selectivity between the groups (*χ* ^2^ = 4.351 (*n* = 91); *p* = 0.049) In relation to mealtime behaviour, statistically significant differences (*p* < 0.05) were observed for all three dimensions studied, i.e., food rejection, disruptive behaviour and limited variety, with higher scores in the ASD group than among the controls Those children with ASD that showed food hyper selectivity (defined as rejection of 33% or more of the offered foods according to the FFQ) also exhibited significantly more food rejection than the TD group (*p* = 0.025)	Tool: AXIS Score: 16/20 Percentage: 80%
Li et al. (2024), China	Design: Cross‐sectional questionnaire Sample size: *n* = 242 Diagnoses: ASD (previous diagnosis) Gender: Male 81%; 67.8% Age: 6.3; 6.8 Ethnicity: Unreported	Food insecurity: Unreported Feeding behaviours: FFQ; SSP; BAMBI	Non‐ASD	Prevalence of feeding behaviours: Children with ASD had poorer diets with fewer vegetables/fruits, less variety of food, a higher degree of inadequate/unbalanced dietary intake, and more severe constipation/total GI symptoms than age‐matched TDC Within the ASD group, compulsive behaviour (a subtype of RRBs) and taste/smell sensitivity were the only traits associated with lower vegetables and fruit consumption, respectively. Self‐injurious behaviour (a subtype of RRBs) was the only contributing trait to less variety of food Limited variety (a subtype of mealtime behaviour problems) and ASD symptom severity were the primary and secondary contributors to inadequate dietary intake, respectively. ASD symptom severity and limited variety were the primary and secondary contributors to unbalanced dietary intake, respectively Notably, unbalanced dietary intake was a significant independent factor associated with constipation/total GI symptoms, and autism‐linked traits manifested no contributions	Tool: AXIS Score: 16/20 Percentage: 80%
Liu et al. (2016), China	Design: Cross‐sectional questionnaire Sample size: *n* = 227 (*n* = 154 ASD) Diagnoses: ASD (diagnosed by CARS) Gender: 208 boys Age: Mean 5.21 Ethnicity: Unreported	Food insecurity: Unreported Feeding behaviours: Self‐created questionnaire	Non‐ASD	Prevalence of feeding behaviours: The ZHA, ZWA and ZBMIA were found to be significantly lower in the children with ASD compared with those without ASD. In addition, the percentages of children exhibiting severe picky eating and severe resistance to new foods, as well as those with a reported general impression of severe eating problems and constipation, were higher among the children with ASD These children consumed significantly fewer macronutrients compared with the children without ASD. In addition, the children with ASD had the highest rate of vitamin A deficiency, followed by iron deficiency. After adjusting for sex, the vitamin A concentration was found to be negatively correlated with the CARS score (*r*s = 0.222, *p* = 0.021). No correlation between the ferritin, folate, vitamin D, or vitamin B12 concentration and the CARS score was found	Tool: AXIS Score: 16/20 Percentage: 80%
Luisier et al. (2019), France	Design: Intervention Sample size: *n* = 49 Diagnoses: ASD (diagnosed by ADOS) Gender: 6 females Age: Mean 104.9 months Ethnicity: Unreported	Food insecurity: Unreported Feeding behaviours: French Adapted Food Neophobia Scale (AFNS) & SSP	None	Prevalence of feeding behaviours: Results revealed a specific increase in positive emotions for the familiarised odour and that 68% of the children chose the food associated with the ‘familiarised odour’ (children who chose the ‘familiarised odour’ food exhibited significantly more sensory particularities)	Tool: MMAT Score: 5/7 Percentage: 71%
Magaña et al. (2023), USA	Design: Cross‐sectional data analysis Sample size: *n* = 124 Diagnoses: ASD (self‐reported) Gender: 100% male Age: 9–10 years Ethnicity: Unknown	Food insecurity: Individual socioeconomic position (SEP) Feeding behaviours: Unreported	None	Prevalence of food insecurity: The racial/ethnic distribution was: 17.1% Black, 14.6% Latino, and 68.3% White; average age was 10 years. Both Black (PR 2.57, 95% CI: 1.26–5.26) and Latino boys (PR 2.08, 95% CI: 1.08–4.03) with ASD were more likely to be obese than their White peers. Although there were significant differences in some social determinants of health by race/ethnicity, only food insecurity mediated associations between race/ethnicity (Black vs. White) and obesity	Tool: AXIS Score: 16/20 Percentage: 80%
Malhi et al. (2017), India	Design: Cross‐sectional questionnaire Sample size: *n* = 113 (*n* = 63 ASD) Diagnoses: ASD (diagnosed by DSM‐5 criteria) Gender: 90.5% male Age: Mean 6.11 years Ethnicity: Unreported	Food insecurity: Unreported Feeding behaviours: CEBI	Non‐ASD	Prevalence of feeding behaviours: The majority (79%) of the parents of children with ASD reported some concern regarding their feeding behaviour as compared to 64% of the parents of TD children As compared to controls, Children with ASD had significantly higher CEBI scores (97.28 vs. 89.48, *t* = 3.15, *p* = 0.002) and more feeding problems (6.42 vs. 2.70, *t* = 3.74, *p* = 0.001) Relative to controls, children with ASD consumed fewer number of food items (*p* = 0.022), particularly fruits (*p* = 0.004), vegetables (*p* = 0.011), and proteins (*p* = 0.015); had significantly lower daily intake of potassium (*p* = 0.001), copper (*p* = 0.007) and folate (*p* = 0.001) Although children with ASD did not differ significantly from controls on intake of calories, height, weight, or body mass index, significantly greater proportion of children with ASD failed to meet the estimated average requirement of thiamine (*p* = 0.039), vitamin C (*p* = 0.013) and copper (*p* = 0.005)	Tool: AXIS Score: 16/20 Percentage: 80%
Matthews et al. (2024), UK	Design: Cross‐sectional survey Sample size: *n* = 351 Diagnoses: ASD (previous diagnosis) Gender: Male 71.2% Age: 9.3 mean Ethnicity: White 91.5%	Food insecurity: Unreported Feeding behaviours: CEBQ; MIOH	ADHD; non‐ASD	Prevalence of feeding behaviours: Neurodevelopmental condition groups reported higher food fussiness, emotional undereating, problematic child mealtime behaviours, dietary concerns, caregiver and spousal stress, and less conventionally structured mealtimes than neurotypical families Attention deficit hyperactivity disorder and Autism spectrum condition + attention deficit hyperactivity disorder groups reported higher food responsiveness, problematic behaviour and caregiver stress than the Autism spectrum condition group Conversely, Autism spectrum condition and Autism spectrum condition + attention deficit hyperactivity disorder groups reported lower food enjoyment and mealtime structure than the attention deficit hyperactivity disorder group	Tool: AXIS Score: 16/20 Percentage: 80%
Mayes and Zickgraf (2019), USA	Design: Cross‐sectional questionnaire Sample size: *n* = 2102 (*n* = 146 ASD) Diagnoses: ASD (diagnosed by CASD) Gender: 78.9% male Age: 1–17 years Ethnicity: 88.8% White	Food insecurity: Unreported Feeding behaviours: Checklist for Autism Spectrum Disorder (CASD)	Other disabilities and non‐ASD	Prevalence of feeding behaviours: Atypical eating behaviours were significantly more common in childrem with ASD (70.4%) than in children with other disorders (13.1%) and typical children (4.8%) For children with ASD who had atypical eating behaviours, the most common behaviour was limited food preferences (88%), followed by hypersensitivity to food textures (46%), other peculiar patterns most often eating only one brand of food (27%), pocketing food without swallowing (19%), and pica (12%) Grain products and/or chicken (usually nuggets) were the preferred foods for 92% of children with ASD who had limited food preferences For children with ASD who had atypical eating behaviours, 25% had three or more atypical eating behaviours (vs. 0% for children with other disorders or typical development). Only children with ASD had pica or pocketed food	Tool: AXIS Score: 16/20 Percentage: 80%
Molina‐López et al. (2021), Spain	Design: Cross‐sectional questionnaire Sample size: *n* = 144 (*n* = 55 ASD) Diagnoses: ASD (diagnosed by DSM‐5 criteria) Gender: Unreported Age: 6–18 years Ethnicity: Unreported	Food insecurity: Unreported Feeding behaviours: FFQ, BAMBIC & 72 h recall	Non‐ASD	Prevalence of feeding behaviours: Results showed a greater presence of children with a low weight (18.4% ASD vs. 3.20% comparison group) and obesity (16.3% ASD vs. 8.6% comparison group) in the ASD group for body mass index (BMI) categories (*p* = 0.003; number needed to take [NNT] = 8.07) The presence of obesity children with ASD compared to the comparison group was even higher when considering the fat component (47.5% ASD vs. 19.4% comparison group, *p* = 0.002; NNT = 10.3). Children with ASD had greater intake inadequacy (50% ASD vs. 22% comparison group, *p* = 0.014; NNT = 3.58), high food selectivity by FFQ (60.6% ASD vs. 37.9% comparison group, *p* < 0.037; NNT = 4.41), and more eating problems (food rejection, limited variety, disruptive behaviour), compared to neurotypical children (*p* = 0.001)	Tool: AXIS Score: 16/20 Percentage: 80%
Munira et al. (2020), Bangladesh	Design: Cross‐sectional questionnaire Sample size: *n* = 32 Diagnoses: ASD (self‐reported) Gender: 65.6% male Age: 9–34 years Ethnicity: Unreported	Food insecurity: Unreported Feeding behaviours: Self‐created questionnaire & 24 h recall	None	Prevalence of feeding behaviours: According to the findings, 90.62% individuals were carrying more weight than the desire weight Among different age groups, the highest mean of carbohydrate intake per day was found in the adolescent group with a standard deviation of 212.54 ± 45.45 g. The lowest mean of fat intake per day was found in the child group with a standard deviation of 25.04 ± 2.79 g	Tool: AXIS Score: 16/20 Percentage: 80%
Nakaoka et al. (2024), Japan	Design: Cross‐sectional questionnaire Sample size: *n* = 256 Diagnoses: ASD (DSM‐5 criteria) Gender: Male (*n* = 96) Age: mean 5 Ethnicity: Unreported	Food insecurity: Unreported Feeding behaviours: ASD‐MBQ	Non‐ASD	Prevalence of feeding behaviours: The mean total and all subdomain scores of the ASD‐MBQ obtained from children with ASD were significantly higher than the scores of those non‐autistic children (*p* < 0.001). The total mean score of the ASD‐MBQ differentiated children with ASD from non‐autistic children with an excellent ROC‐AUC of 0.885 (95% CI 0.845–0.942). A total mean score of 2.04, calculated using the Youden score showed the best sensitivity (0.648) and specificity (0.953)	Tool: AXIS Score: 16/20 Percentage: 80%
Önal and Uçar (2024), Turkey	Design: Cross‐sectional questionnaire Sample size: *n* = 70 Diagnoses: ASD (previous diagnosis) Gender: Male (*n* = 49) Age: mean 12 Ethnicity: Unreported	Food insecurity: Unreported Feeding behaviours: BPFAS	None	Prevalence of feeding behaviours: The frequency of obesity in the males with ASD was higher than that in the females with ASD. BPFAS scores were higher in the females than in the males for all frequencies (total score, total problem score, child total score, child problem, parent total score, and parent problem). BPFAS total score was higher in the underweight children group (especially in the females) (*p* < 0.05)	Tool: AXIS Score: 16/20 Percentage: 80%
Panjwani et al. (2021a), USA	Design: Cross‐sectional survey Sample Size: *n* = 200 Diagnosis: ASD (self‐reported) Gender: 76% male Age: 2–17 years Ethnicity: Non‐Hispanic white (62%), 38% unreported	Food insecurity: Validated two‐item screening tool (22 hager) Feeding behaviours: Unreported	None	Prevalence of food insecurity: Food insecurity pre: OR 1.7, 95% CI = 0.96–3 and *p*: 0.01 and post COVID: OR 2.6, 95% CI = 1.45–4.67 and *p* < 0.001 Prevalence of feeding behaviours: Shelter regulations significantly contributed to adverse eating behaviours (OR: 2.30; 95% CI: 1.12–4.72, *p* = 0.03)	Tool: AXIS Score: 17/20 Percentage: 85%
Panjwani et al. (2021b), USA	Design: Cross‐sectional survey Sample size: *n* = 200 Diagnoses: ASD (self‐reported) Gender: 76.1% male Age: 2–17 years Ethnicity: White (62.1%), Black (7.2%), Hispanic (13.9%), mixed (9.7%) and other (7.2%)	Food insecurity: Self‐created survey Feeding behaviours: Self‐created survey	None	Prevalence of feeding behaviours/food insecurity: A majority of respondents reported a moderate‐to‐large impact on the child's overall behaviour (74%) due to COVID‐19 Stratifying by income level and food security status revealed disparities in the impact on overall behaviour and most specific behaviours. Compared to a household income ≥ $100 K, an income <$50 K was associated with an increased risk of moderate‐to‐large impact on the child's overall behaviour (odds ratio (OR): 4.07, 95% CI: 1.60, 10.38). Food insecurity also significantly impacted this risk, even after adjusting for potential confounding factors (OR: 3.31, 95% CI: 1.13, 9.66)	Tool: AXIS Score: 14/20 Percentage: 70%
Park et al. (2020), Korea	Design: Cross‐sectional questionnaire Sample size: *n* = 130 Diagnoses: ASD (recruited from ASD organisations) Gender: 82.3% male Age: 10–21 years Ethnicity: Unreported	Food insecurity: Unreported Feeding behaviours: BAMBI, Swedish Eating Assessment for Autism Spectrum Disorder (SWEAA), FPQ, & SSP	None	Prevalence of feeding behaviours: The highest proportion of students (48.5%) had an average meal duration of ‘15–30 min’, followed by 39.2% who finished meals ‘within 15 min’ and 12.3% with a meal duration of ‘30–40 min’ For the average amount of food consumed at meals over the previous six months, ‘eat a lot (eat until full)’ was the most common response (45.4%), followed by ‘eat moderate’ (35.4%), ‘food intake cannot be controlled, so it must be adjusted’ (13.8%), and ‘eat less’ (5.4%) Overall, the most preferred food groups were meat, fish, eggs, and beans (3.73 ± 1.13); milks and dairy products (3.63 ± 1.19); and snacks (3.54 ± 1.24), whereas the least favourite food groups were fats and sweets (3.18 ± 1.04) and vegetables, seafood, and fruits (3.28 ± 1.19)	Tool: AXIS Score: 15/20 Percentage: 75%
Postorino et al. (2015), Italy	Design: Cross‐sectional questionnaire Sample size: *n* = 158 Diagnoses: ASD (diagnosed by DSM‐5 criteria) Gender: 136 boys Age: 3–12 years Ethnicity: 100% Caucasian	Food insecurity: Unreported Feeding behaviours: FFQ & YAQ	None	Prevalence of feeding behaviours: Overall, the FS (food selectivity) group showed significantly higher rates of ASD symptoms as compared to the No FS group in the questionnaires completed by parents Furthermore, parents of the FS group reported significantly higher levels of parental stress and a larger degree of their children's behavioural problems as compared to the No FS group. Finally, there were no differences between the FS and the No FS group on any adaptive skill domain	Tool: AXIS Score: 15/20 Percentage: 75%
Postorino et al. (2017), Italy	Design: Cross‐sectional questionnaire Sample size: *n* = 57 Diagnoses: ASD (diagnosed by ADOS‐2) Gender: 30 females Age: 10–17 years Ethnicity: Unreported	Food insecurity: Unreported Feeding behaviours: Eating Attitude Test‐26 (EAT‐26) & Eating Disorder Invenory‐3 (EDI‐3)	None	Prevalence of feeding behaviours: Of the 30 AN participants, only three scored above the conventional ADOS‐2 threshold for ASD. The AN participants were similar to their controls on autistic trait measures, and to the ASD group on obsessive‐compulsive measures, and on theory of mind ability and affect recognition measures	Tool: AXIS Score: 18/20 Percentage: 90%
Rababah et al. (2024), Jordan	Design: Cross‐sectional questionnaire Sample size: *n* = 37 Diagnoses: ASD (previous diagnosis) Gender: Male 76% Age: 3–18 years Ethnicity: Unreported	Food insecurity: Unreported Feeding behaviours: FFQ	None	Prevalence of feeding behaviours: Omega‐3 PUFA and omega‐6 PUFA mean intakes were 0.31 ± 0.29 and 5.15 ± 2.91 g/day, respectively. The results indicate that there is no significant difference in most autism scale items with omega‐3 PUFA intake. Omega‐6 PUFA was found to have a significant association with several scale items (*p*‐value < 0.05). Moreover, we found that foods high in omega‐3 PUFA (g/100 g) were walnuts (9.20), tuna (0.92), and sardine (0.90). Foods high in omega‐6 PUFA were sunflower oil (63.3), corn oil (56.0) and soybean oil (53.0)	Tool: AXIS Score: 15/20 Percentage: 75%
Rashid et al. (2021), Pakistan	Design: Cross‐sectional questionnaire Sample size: *n* = 68 Diagnoses: ASD (self‐reported) Gender: 67.6% male Age: 2–11 years Ethnicity: Unreported	Food insecurity: Unreported Feeding behaviours: Self‐created questionnaire	None	Prevalence of feeding behaviours: Out of 68 children, 55 (80.8%) liked rice, 40 (58.8%) liked junk food, 41 (60.2%) liked bread, 38 (55.8%) liked crunchy food and 36 (53%) liked fruits. Out of 68 children 32 (47%) didn't need certain silverware or temperature to eat food, 33 (48%) didn't prefer to sit with specific person or chair at meals, 31 (0.5%) didn't show any behaviour like crying and screaming, 30 (44.1%) didn't refuse food if not in a presentable way and 30 (44.1%) disagreed to eat single food three times	Tool: AXIS Score: 15/20 Percentage: 75%
Riccio et al. (2018), Italy	Design: Cross‐sectional questionnaire Sample size: *n* = 84 (*n* = 43 ASD) Diagnoses: ASD (diagnosed by DSM‐5 criteria) Gender: 33 males Age: 2–11 years Ethnicity: Unreported	Food insecurity: Unreported Feeding behaviours: Feeding Habits Questionnaire (FHQ) & Food Preference Inventory (FPI)	Non‐ASD	Prevalence of feeding behaviours: A statistically significant correlation between PROP sensitivity and food refusal was observed. Furthermore, a prevalence of the PAV‐sensitive haplotype compared to the AVI insensitive one was seen in children with ASD with food selectivity In particular the children with ASD eat on average 30% less vegetables, bitter greens, and fruits, compared to TD ones	Tool: AXIS Score: 18/20 Percentage: 90%
Sanchez‐Cerezo et al. (2024), UK	Design: Observation surveillance Sample size: *n* = 319 Diagnoses: ASD (DSM‐5 criteria) Gender: Female 45.5% Age: 11.2 mean Ethnicity: White 77.7%	Food insecurity: Unreported Feeding behaviours: Self‐created questionnaire	None	Prevalence of feeding behaviours: LCA revealed four distinct classes which were labelled as Fear subtype, Lack of Interest subtype, Sensory subtype, and Combined subtype. The probability of being classified as these were 7.2% (*n* = 23), 25.1% (*n* = 80), 29.5% (*n* = 94) and 38.2% (*n* = 122), respectively. Age at diagnosis, sex, weight loss, distress associated with eating, and autism spectrum disorder diagnosis were identified as predictors of class membership	Tool: MMAT Score: 7/7 Percentage: 100%
Şengüzel et al. (2021), Turkey	Design: Cross‐sectional questionnaire Sample size: *n* = 46 Diagnoses: ASD (clinically confirmed) Gender: 82.6% male Age: 2–10 years Ethnicity: Unreported	Food insecurity: Unreported Feeding behaviours: BAMBI & FFQ	None	Prevalence of feeding behaviours: The rates of being overweight and obese were 10.9% and 28.3%, respectively Food selectivity was observed in 84.8% of the children, and BAMBI food refusal scores were significantly higher for those aged between 2 and 5 years (*p* ¼ 0.03). Autism scores and consumption of milk, yogurt, oily seeds, rice/pasta, and fruits (*p* < 0.05) were significantly correlated. There were also significant differences between these scores and the frequency of consuming eggs, legumes, and other cereals (*p* < 0.05)	Tool: AXIS Score: 18/20 Percentage: 90%
Sharp et al. (2014), USA	Design: RCT Sample size: *n* = 19 Diagnoses: ASD (diagnosed by SRS) Gender: 80% male Age: 3–8 years Ethnicity: Unreported	Food insecurity: Unreported Feeding behaviours: FPI & BAMBI	None	Prevalence of feeding behaviours: Upon completion of the Autism MEAL Plan, caregivers in the treatment group reported a significant reduction (*F* (1, 16) = 7.6, *p* = 0.01) in PSI scores compared to the waitlist control group after controlling for preintervention PSI levels. The magnitude of this effect fell in the large range by conventional standards (*d* = 1.1). There were no significant changes detected in terms of feeding behaviours, as captured by the BAMBI total score and the three BAMBI subscales, or dietary variety based on the FPI selectivity score	Tool: MMAT Score: 5/7 Percentage: 71%
Sharp et al. (2018), USA	Design: Cross‐sectional data analysis Sample size: *n* = 70 Diagnoses: ASD (clinically diagnosed) Gender: 80% male Age: 2–17 years Ethnicity: Non‐hispanic (80%)	Food insecurity: Unreported Feeding behaviours: 3‐day food diary	None	Prevalence of feeding behaviours: Caregivers reported 67% of the sample (*n* ¼ 47) omitted vegetables and 27% omitted fruits (*n* ¼ 19). Seventy‐eight percent consumed a diet at risk for five or more inadequacies. Risk for specific inadequacies included vitamin D (97% of the sample), fibre (91%) vitamin E (83%), and calcium (71%). Children with five or more nutritional inadequacies (n¼55) were more likely to make negative statements during meals (*p* < 0.05). Severe food selectivity was not associated with compromised growth or obesity	Tool: AXIS Score: 14/20 Percentage: 70%
Smith et al. (2020), England	Design: Cross‐sectional questionnaire Sample size: *n* = 98 (*n* = 27 ASD) Diagnoses: ASD (self‐reported) Gender: 22 males Age: Mean 10.4 years Ethnicity: Unreported	Food insecurity: Unreported Feeding behaviours: FPQ, CEBQ, & SSP	Other disorders and non‐ASD	Prevalence of feeding behaviours: Children with neurodevelopmental disorders were reported to have significantly higher levels of both food fussiness and sensory sensitivity, with children with ASD and TS also showing significantly less preference for fruit than children with TD Importantly, higher levels of taste/smell sensitivity predicted food fussiness for all four groups of children. In addition, taste/smell sensitivity fully mediated the differences in food fussiness between each group of neurodevelopmental disorders compared to the TD group	Tool: AXIS Score: 16/20 Percentage: 80%
Son and Alford (2024), USA	Design: Intervention Sample size: *n* = 15 Diagnoses: ASD (previous diagnosis) Gender: Male 80% Age: mean 13 Ethnicity: Korean 100%	Food insecurity: Unreported Feeding behaviours: USDA MyPlate	None	Prevalence of feeding behaviours: The findings of the study suggest that the pilot program was effective in gaining nutrition knowledge and increasing physical involvement among participants	Tool: MMAT Score: 6/7 Percentage: 86%
St. John and Ausderau (2024), USA	Design: Cross‐sectional survey Sample size: *n* = 427 Diagnoses: ASD (previous diagnosis) Gender: Male 82.9% Age: 8.42 mean Ethnicity: White 88.52%	Food insecurity: Unreported Feeding behaviours: Survey for Characterisation of Feeding Challenges in Autistic Children—United States; FEAST	None	Prevalence of feeding behaviours: Sensory‐based feeding challenges were the most common. Age of onset of feeding challenges was significantly lower than the age of autism diagnosis. Early feeding challenges differentially predicted feeding challenge severity and classification scores	Tool: AXIS Score: 15/20 Percentage: 75%
Suarez and Crinion (2015), USA	Design: Cross‐sectional questionnaire Sample size: *n* = 54 Diagnoses: ASD (self‐reported) Gender: 88% male Age: 55–128 months Ethnicity: 96% Caucasian	Food insecurity: Unreported Feeding behaviours: FFQ	None	Prevalence of feeding behaviours: Children who had 20 or less foods in their diet repertoire had a significantly lower % vegetables and fruits, and a significantly higher percentage of dairy and grain/potato/snack foods than children with more than 20 foods as part of their regular diet Also, children with 20 or less foods had a greater % empty calories in total diet compared to children who had > 20 foods	Tool: AXIS Score: 17/20 Percentage: 85%
Tahech et al. (2023), USA	Design: Cross‐sectional data analysis Sample size: *n* = 59 725 (*n* = 1702 ASD) Diagnoses: ASD (self‐reported) Gender: 79% male Age: Mean 10.44 years Ethnicity: White (47.90%) Black (15.35%), Hispanic (27.31) and non Hispanic (9.44%)	Food insecurity: NSCH Feeding behaviours: National Survey of Children's Health	Non‐ASD	Prevalence of feeding behaviours/food insecurity: A greater percentage of parents of children with ASD reported weight‐related concerns about their child (*p* < 0.001), food insecurity (*p* < 0.001), and fewer family meals together (*p* = 0.04) compared to parents of NT youth. Results from the regression analysis revealed that the odds of weight concerns for youth with ASD were 2.29 times (95% CI = 1.62–3.25) the odds of weight concerns for NT youth	Tool: AXIS Score: 18/20 Percentage: 90%
Tanner and Andreone (2015), Canada	Design: Case study Sample size: *n* = 1 Diagnoses: ASD (previous diagnosis) Gender: 100% male Age: 3 years Ethnicity: Unreported	Food insecurity: Unreported Feeding behaviours: BAMBI	None	Prevalence of feeding behaviours: A preference assessment, parent interview and food journal determined the child's food repertoire consisted of four different foods in total (pasta, fish crackers, dry cereal and yogurt), and the child was selective by brand, texture, temperature and utensil requirement After 9 months of treatment, the participant's food repertoire increased from four items to more than 50 items. Additionally, food refusal behaviour decreased to rates of zero during intervention and parents report significant decreases in mealtime behaviour at home	Tool: MMAT Score: 7/7 Percentage: 100%
Tanner and Andreone (2015), USA	Design: Cross sectional questionnaire Sample size: *n* = 35 Diagnosis: ASD (self‐reported) Gender: 32 males Age: 4–10 years Ethnicity: White (*n* = 22), Non‐White (*n* = 13)	Food insecurity: Validated two‐item screening tool (22 hager) Feeding behaviours: BAMBIC	None	Prevalence of food insecurity: Selective eating group: food secure (*n* = 13) and insecure (*n* = 4). Non‐selective eating group: food secure (*n* = 12), insecure (*n* = 6) Prevalence of feeding behaviours: Selective eating group ate fewer total foods (*p* < 0.001) Selective eating group had a higher rate of food refusal (*p* < 0.001) Significant relationship between the food refusal subscale and the limited variety, *r* = 0.486, *p* < 0.01 scale of the BAMBIC Significant relationship between SSP taste/smell sensitivity scores and total foods eaten, *r* = 0.363, *p* < 0.05 Significant relationships between SSP taste/smell sensitivity scores and food refusal, *r* = −0.419, *p* < 0.01, scores on the BAMBIC	Tool: AXIS Score: 16/20 Percentage: 80%
Thullen and Bonsall (2017), USA	Design: Cross‐sectional survey Sample size: *n* = 113 Diagnoses: ASD (recruited from ASD organisations) Gender: 81.3% male Age: 5–13 years Ethnicity: Unreported	Food insecurity: Unreported Feeding behaviours: BAMBI	None	Prevalence of feeding behaviours: Results indicated that food selectivity was both the most frequently reported type of challenging feeding behaviour and the most often reported as problematic but was also the only type of challenging feeding behaviour that was not associated with parenting stress. Child disruptive behaviour at mealtime was the only feeding challenge associated with quality of co‐parenting	Tool: AXIS Score: 15/20 Percentage: 75%
Cui et al. (2024), China	Design: Cross‐sectional questionnaire Sample size: *n* = 82 Diagnoses: ASD (from special education school) Gender: Male 82.4% Age: 11.8 mean Ethnicity: Unreported	Food insecurity: Unreported Feeding behaviours: SP; 3‐day food diary	None	Prevalence of feeding behaviours: Overall SSP score was not statistically correlated with the number of inadequate nutrients, but SSP subscale was linked to nutrient intake. Poor nutrition status, minerals, and vitamins were detected	Tool: AXIS Score: 15/20 Percentage: 75%
Tomova et al. (2020), Slovakia	Design: Cross‐sectional questionnaire Sample size: *n* = 62 (*n* = 46 ASD) Diagnoses: ASD (diagnosed by DSM‐5 criteria) Gender: 100% male Age: 4–8 years Ethnicity: Unreported	Food insecurity: Unreported Feeding behaviours: FFQ	Non‐ASD	Prevalence of feeding behaviours: Of the 67 children, 28.4% had feeding difficulties (high BPFAS total frequency score). Caregiver feeding styles were authoritarian (34.8%) or indulgent (39.4%) Child characteristics did not significantly predict for the severity of feeding difficulties. Univariate analysis revealed that authoritarian feeding style (*p* Z 0.001) and ABC hyperactivity score (*p* Z 0.006) were significantly associated with BPFAS severity score A final regression model including all child characteristics and ABC scores did not reveal any significant predictors of BPFAS total frequency score (*R* ^2^ Z 0.557)	Tool: AXIS Score: 15/20 Percentage: 75%
Van Der Lubbe et al. (2024), Netherlands	Design: Cross‐sectional questionnaire Sample size: *n* = 222 Diagnoses: ASD (diagnosed by ADOS‐2) Gender: Male 82.1% Age: 3–7 years Ethnicity: Dutch 64.4%	Food insecurity: Unreported Feeding behaviours: CBEQ	None	Prevalence of feeding behaviours: Children with ASD were 8 times more often obese (16.8%) than children from the general population (2.0%). Child BMI correlated positively with child food approach behaviour and maternal BMI, and correlated negatively with child ‘Slowness in eating’. There was no correlation between child BMI and ASD severity, problem behaviour, parental eating behaviour, parental stress and SES	Tool: AXIS Score: 16/20 Percentage: 80%
Wallace et al. (2018), England	Design: Cross‐sectional questionnaire Sample size: *n* = 4564 (*n* = 37 ASD) Diagnoses: ASD (diagnosed by CAST) Gender: 33 males Age: 8–11 years Ethnicity: Unreported	Food insecurity: Unreported Feeding behaviours: CFNS	Non‐ASD	Prevalence of feeding behaviours: Children with ASD were rated as more food neophobic than their same‐age non‐ASD peers (2.67 ± 0.83 vs. 2.22 ± 0.73; *p* < 0.001) and there were subclinical associations between FN and ASD traits (social, communication, and restricted/repetitive behaviour) in this community‐based sample of children Moreover, whereas FN alone predicted lower BMI, the interaction of FN and ASD traits predicted higher BMI, suggesting that elevated ASD traits in combination with FN exert opposing influences on weight compared with FN alone	Tool: AXIS Score: 16/20 Percentage: 80%
Wenzell et al. (2024), USA	Design: Cross‐sectional questionnaire Sample size: *n* = 103 Diagnoses: ASD (diagnosed by Autism Diagnostic Observation Schedule‐Second Edition or CARS‐2) Gender: Male 77.7% Age: 5.8 mean Ethnicity: Black 54.5%	Food insecurity: Unreported Feeding behaviours: BAMBI and 24‐h recall	None	Prevalence of feeding behaviours: Of 103 children, 45.6% (*n* = 47) were classified as FS; 54.4% (*n* = 56) no FS. After adjusting for potential confounders, the odds of FS increased by 1.91 (95% CI: 1.38, 2.64, *p* < 0.001) for every half‐SD increase in BAMBI total score and by 1.35 (95% CI: 1.05, 1.74, *p* = 0.020) for every half‐SD increase in ABC hyperactivity/noncompliance. No group differences in anthropometrics or nutritional intake were identified	Tool: AXIS Score: 15/20 Percentage: 75%
Xie et al. (2024), China	Design: Cross‐sectional questionnaire Sample size: *n* = 160 Diagnoses: ASD (previous diagnosis) Gender: Male 78.8% Age: 4.23 mean Ethnicity: Unreported	Food insecurity: Unreported Feeding behaviours: CFNS; CFQ; FFQ	None	Prevalence of feeding behaviours: The mean FN score was 25.56 ± 6.46. The caregiver's pressure to eat positively related to children's FN (*β* = 0.164 95% CI, 0.078, 2.163). In these children, a negative correlation between FN score and the frequency of vegetable intake (*p* ≤ 0.001), fruit intake (*p* ≤ 0.05), aquatic product intake (*p* ≤ 0.05), and dietary diversity score (*p* ≤ 0.01) were found, and positively correlated with the frequency of snack intake (*p* ≤ 0.05)	Tool: AXIS Score: 15/20 Percentage: 75%
Zickgraf and Mayes (2018), USA	Design: Cross‐sectional questionnaire Sample size: *n* = 1112 Diagnoses: ASD (clinical diagnosis) Gender: 81.8% male Age: 1–17 years Ethnicity: 89.4% White	Food insecurity: Unreported Feeding behaviours: CASD	None	Prevalence of feeding behaviours: Atypical eating behaviours were found in 70.5% of children and were positively related to only four variables: age (most common at ages 1–3), increasing autism severity (total number of autism symptoms present), poor appetite, and constipation	Tool: AXIS Score: 17/20 Percentage: 85%
Zobel‐Lachiusa et al. (2015), USA	Design: Cross‐sectional questionnaire Sample size: *n* = 68 (*n* = 34 ASD) Diagnoses: ASD (self‐reported) Gender: 33 males Age: 5–12 years Ethnicity: 85% White	Food insecurity: Unreported Feeding behaviours: SSP, BAMBI, & Sensory Eating Checklist (SEC)	Non‐ASD	Prevalence of feeding behaviours: Results from parent‐report and child‐report questionnaires indicated that children with ASD scored significantly differently from TD peers on the measures of sensory differences and eating behaviours. Data also supported a correlation between sensory differences and eating difficulties in children with ASD	Tool: AXIS Score: 15/20 Percentage: 75%

Abbreviations: ADHD, Attention Deficit Hyperactivity Disorder; ADI‐R, Autism Diagnostic Interview–Revised; ADOS, Autism Diagnostic Observation Schedule; ADOS‐2, Autism Diagnostic Observation Schedule, Second Edition; AFIS, Autism Food Insecurity Survey; AN, Anorexia Nervosa; aOR, Adjusted Odds Ratio; ARFID, Avoidant/Restrictive Food Intake Disorder; ASD, Autism Spectrum Disorder; ASFIS, Autism Speaks Food Insecurity Survey; AXIS, Appraisal Tool for Cross‐Sectional Studies; BAMBI/BAMBIC, Brief Autism Mealtime Behaviour Inventory; BMI, body mass index; BPFAS, Behavioural Paediatric Feeding Assessment Scale; CARS/CARS‐2, Childhood Autism Rating Scale; CASD, Checklist for Autism Spectrum Disorder; CAST, Childhood Autism Spectrum Test; CEBI, Children's Eating Behaviour Inventory; CEBQ, Child Eating Behaviour Questionnaire; CFNS, Child Food Neophobia Scale; CFQ, Child Feeding Questionnaire; CFSQ, Caregiver Feeding Style Questionnaire; CI, confidence interval; COVID‐19, coronavirus disease 2019; CPEBQ, Children's Parent Eating Behaviour Questionnaire; DSM‐5, Diagnostic and Statistical Manual of Mental Disorders, Fifth Edition; FFQ, Food Frequency Questionnaire; FPQ, Food Preference Questionnaire; FV, fruit and vegetables; GI, gastrointestinal; HEI, Healthy Eating Index; HPS, Household Pulse Survey; ICD‐10, International Classification of Diseases, Tenth Revision; ID, intellectual disability; MIOH, Meals in Our Household Questionnaire; MMAT, Mixed Methods Appraisal Tool; NNT, number needed to treat; NSCH, National Survey of Children's Health; NSCHCN, National Survey of Children with Special Health Care Needs; OR, odds ratio; PFSQ, Parental Feeding Style Questionnaire; PR, prevalence ratio; RAISD, Reinforcer Assessment for Individuals with Severe Disabilities; RCT, randomised controlled trial; RRR, relative risk ratio; SCDC, Social and Communication Disorders Checklist; SD, standard deviation; SEP, socioeconomic position; SEQ, Sensory Experiences Questionnaire; SES, socioeconomic status; SHCN, Special Health Care Needs; SNAP, Supplemental Nutrition Assistance Program; SP, Sensory Profile; SRS/SRS‐2, Social Responsiveness Scale; SSP/SSP2‐2, Short Sensory Profile; STEP‐CHILD, Screening Tool of Feeding Problems for Children; TD, typically developing; TDC, typically developing children; UAE, United Arab Emirates; UK, United Kingdom; USA, United States of America.

### 2.6 Analysis

Owing to substantial methodological heterogeneity across the included studies including differences in study design, participant characteristics, outcome measures and reporting of feeding behaviours, statistical meta‐analysis was not considered appropriate. Instead, a narrative synthesis was undertaken in accordance with the guidance of Popay et al. (2006), alongside qualitative content analysis (Krippendorff 2018; Finfgeld‐Connett 2013).

Following data extraction, all findings relating to feeding behaviours were imported into a structured analytical framework developed in Microsoft Excel. Data from each study were systematically charted according to study characteristics (e.g., country, design, sample, participant characteristics, diagnostic approach, feeding measures, intervention where applicable and key findings) together with all reported feeding‐related outcomes. This process enabled direct comparison of findings across studies despite methodological differences.

The extracted findings were then profiled by comparing similarities and differences in reported feeding behaviours, associated factors and intervention outcomes. Two reviewers (E.T. and B.H.) independently read the extracted findings repeatedly to become familiar with the data before undertaking inductive coding. Individual findings were assigned descriptive codes that captured the primary feeding‐related concepts reported within each study (e.g., food selectivity, food refusal, sensory sensitivity, nutritional deficiencies, mealtime behaviours, environmental influences and intervention outcomes).

Codes describing conceptually similar findings were iteratively clustered into broader descriptive categories through constant comparison across studies. These categories were subsequently refined through discussion between the reviewers to generate higher‐order themes that represented recurring patterns across the evidence base. Themes were reviewed against the original extracted data to ensure they accurately reflected the findings reported within the included studies. Any disagreements regarding coding or theme development were resolved through discussion until consensus was reached.

To enhance transparency, the frequency with which each theme appeared across studies was recorded and presented descriptively. Frequency counts were used only to indicate how commonly particular feeding behaviours or associated factors were reported and were not interpreted as measures of importance or effect size. Less frequently reported findings were retained where they contributed meaningful insights to the evidence base.

The qualitative and mixed‐methods studies were analysed separately using conventional content analysis. Meaning units describing feeding experiences, mealtime practices, parental perspectives and support needs were identified, coded and grouped into higher‐order codes through an iterative process. These codes were then synthesised into overarching themes representing shared experiences across studies. Finally, findings from the narrative synthesis and content analysis were integrated to provide a comprehensive interpretation of the quantitative and qualitative evidence.

To improve consistency across the heterogeneous evidence base, extracted findings were organised into broad analytical domains reflecting the primary outcomes reported within the included studies. These domains included feeding behaviours (e.g., food selectivity, food refusal and sensory‐related feeding difficulties), nutritional outcomes (e.g., dietary diversity, nutritional status and weight‐related outcomes), demographic and environmental factors associated with feeding behaviours and the effectiveness of feeding interventions. Within each domain, findings were synthesised narratively, whereas allowing additional sub‐themes to emerge inductively during the analytical process.

## 3 Results

**FIGURE 1:**
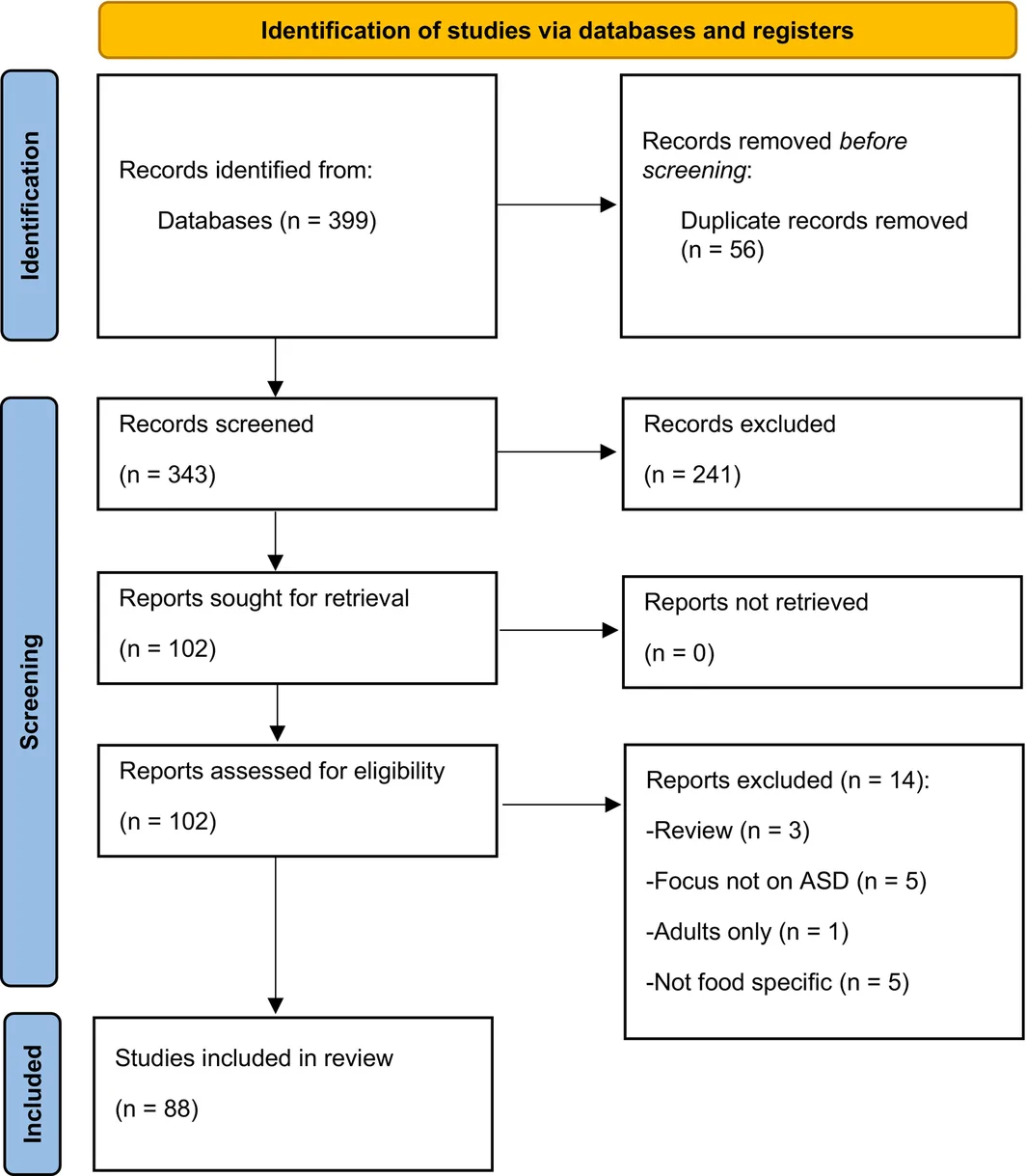
PRISMA diagram (Page et al. 2021).

The PRISMA flow diagram is shown in Figure 1. The initial searches identified 399 records. After de‐duplication (*n* = 56 total removed) and title and abstract sifting (*n* = 241 removed), 102 full papers were assessed. In total, 88 papers met the inclusion criteria and were included in the review. The articles were published between 2013 and 2024. The reasons for exclusions are documented in Figure 1.

### 3.1 Study Characteristics

The characteristics from the 88 included papers are presented in Table 1 (see table for references to each study). Seventy‐three papers (83%) used a quantitative design, eight papers (9%) used a qualitative design and seven papers (8%) used mixed methods. Thirty‐one papers (35%) included comparators: autism and non‐autism (*n* = 27), autism and intellectual disability (ID) (*n* = 1) and autism, other disabilities and non‐ASC (*n* = 3).

Thirty‐three papers (37.5%) reported on research from the US, nine (10%) from England, six (7%) from Turkey, five (6%) from Italy and China, three (3%) from Spain and India, two (2%) each from Bangladesh, Brazil, Japan, Pakistan and Poland and one (1%) each from Canada, France, Ireland, Jordan, Korea, Malaysia, Netherlands, Oman, Philippines, Saudi Arabia, Singapore, Slovakia, South Africa and UAE.

### 3.2 Participant Characteristics

The total sample size was 87 320, with 36 983 being unique children with autism. The mean percentage of males across all papers was approximately 75.4%, and the mean age was approximately 6.23 years, with the range being between 0 and 25 years. Thirty‐six papers reported participant ethnicity; mean percentage White/Caucasian (60%) and Black (16%) participants.

### 3.3 Autism Diagnosis

Studies used different methods of confirming autism diagnosis. The most commonly used tools to identify autism included the Diagnostic and Statistical Manual of Mental Disorders (DSM‐5) (Regier et al. 2013) criteria (*n* = 21), self‐reports (*n* = 19) and studies reporting a confirmed clinical diagnosis of autism made by a qualified healthcare professional (*n* = 19). However, these studies did not consistently specify the diagnostic procedures or standardised assessment tools used to establish the diagnosis. Other tools included: assumed by membership of autism organisations (*n* = 5), Autism Diagnostic Interview‐Revised (ADI‐R) (*n* = 4), Autism Diagnostic Observation Schedule (ADOS‐2) (*n* = 6), assumed by attendance at autism schools (*n* = 3), Childhood Autism Rating Scale (CARS) (*n* = 4), Checklist for Autism Spectrum Disorder (CASD) (*n* = 1), Childhood Autism Spectrum Test (CAST) (*n* = 1), the International Classification of Diseases (ICD‐10) (*n* = 1), the Social and Communication Disorder Checklist (SCDC) (*n* = 1), Social Responsiveness Scale (SRS) (*n* = 1), Social Skills Improvement System (SSiS) (*n* = 1) and one study did not state the tool used.

### 3.4 Feeding Behaviours

A wide range of assessment methods were used across studies. Standardised questionnaires were the most common approach including validated measures of feeding behaviour, dietary intake, sensory processing and parent feeding practices (e.g., FFQ, BAMBI, SSP and CEBQ). However, several studies used researcher‐developed or otherwise non‐validated questionnaires, resulting in considerable variation in the assessment approaches employed. Dietary intake was also assessed using food records and recall methods (e.g., 24‐h recalls and food diaries), whereas a small number of studies used qualitative interviews or focus groups or evaluated feeding interventions rather than questionnaire‐based assessments. A complete list of assessment measures is provided in Table S1.

### 3.5 Methodological Quality

Critical appraisal scores for each study are reported in Table 1. Overall study quality was medium to high across both tools. For studies appraised with AXIS, compliance ranged from 65% to 100%, with the vast majority (*n* = 53) scoring between 75% and 80%. For studies appraised with MMAT, scores ranged from 71% to 100%, with over half (*n* = 11, 55%) achieving the maximum score of 100%.

Overall, the methodological quality of the included studies was moderate to high. Across the AXIS assessments, studies consistently demonstrated clear research aims, appropriate study designs, well‐defined target populations, valid outcome measures and conclusions that were supported by the reported findings. The most common methodological limitations related to inadequate justification of sample size, limited reporting of non‐response bias and non‐responders and incomplete reporting of funding sources or potential conflicts of interest. The MMAT assessments similarly indicated generally good methodological quality across the qualitative, mixed‐methods and intervention studies, with most studies demonstrating appropriate methodologies and findings supported by the data. However, some studies were limited by issues relating to participant representativeness, attrition, confounding or insufficient justification of methodological approaches. These findings highlight the need for future research to improve reporting transparency, justify sample size decisions, minimise attrition where possible and strengthen the reporting of participant recruitment and response characteristics.

### 3.6 Narrative Synthesis

Eighty‐seven studies were narratively synthesised, comprising 78 quantitative, two qualitative and seven mixed‐methods studies. Where studies included comparison groups (e.g., neurotypical children or children with other developmental conditions), this is explicitly stated in the synthesis. Studies without comparison groups report findings for autistic participants only.

#### 3.6.1 Food Selectivity

Food selectivity refers to a restricted pattern of food intake characterised by acceptance of a limited variety of foods or rejection of particular foods based on characteristics such as taste, texture, colour, smell, temperature or presentation (Bandini et al. 2010; Cermak et al. 2010). Although food selectivity may include food refusal, the two concepts are distinct. Food refusal refers to the rejection of specific foods or meals, whereas food selectivity encompasses the broader pattern of restricted dietary variety and preferences. Throughout this review, food selectivity is discussed separately from food refusal where studies distinguished between these constructs.

Across 35 studies, food selectivity emerged as one of the most frequently reported feeding behaviours among children with autism. Studies consistently reported preferences for starchy foods and snack foods, alongside reduced consumption of protein‐rich foods (Adams et al. 2021; Amin et al. 2022; Attlee et al. 2015; Huxham et al. 2019; Rashid et al. 2021). Additional feeding characteristics included preferences for particular brands, packaging, food presentation and mealtime routines, with some children requiring distraction during meals or parental assistance with feeding (Huxham et al. 2019; Kazek et al. 2021). In studies without comparison groups, food selectivity was frequently reported as one of the most common caregiver‐identified feeding concerns, affecting up to 81.7% of participants (Leader et al. 2021).

Dietary diversity was examined separately from food selectivity in several studies. Although closely related, dietary diversity refers to the variety of foods consumed rather than the behavioural preference for particular foods. Across these studies, greater food selectivity was consistently associated with lower dietary diversity, poorer overall diet quality, reduced protein intake and increased risk of nutritional deficiencies. Several studies also reported inadequate intake in vitamin D, calcium and other micronutrients, together with increased gastrointestinal concerns among children with more selective eating patterns (Hümeyra İslamoğlu et al. 2024; Islam et al. 2024; Li et al. 2024; Cui et al. 2024; Rababah et al. 2024; Wenzell et al. 2024). However, one study found no significant differences in anthropometric measures or overall nutrient intake between selective and non‐selective eaters (Wenzell et al. 2024).

Across studies comparing children with autism and neurotypical children, children with autism consistently demonstrated greater food selectivity, consumed fewer food groups, particularly fruits and vegetables and exhibited higher rates of restricted eating behaviours and micronutrient inadequate intake (Bandini et al. 2010; Hubbard et al. 2014; Postorino et al. 2015; Tanner and Andreone 2015). Several studies also reported that greater food selectivity was associated with increased autism severity, greater reluctance to try unfamiliar foods and narrower food repertoires (Adams et al. 2021; Mayes and Zickgraf 2019; Postorino et al. 2015). Although dietary variety generally increased with age, food selectivity remained common throughout childhood, particularly among children with more severe autistic characteristics (Adams et al. 2021; Bandini et al. 2010; Zickgraf and Mayes 2018).

#### 3.6.2 Food Sensitivity and Sensory Issues

Fourteen studies examined sensory processing and its relationship with feeding behaviours. Across both studies involving children with autism only and studies comparing children with autism and non‐autistic children, sensory hypersensitivity was consistently associated with greater food selectivity, food refusal, food fussiness and atypical eating behaviours. Taste, smell and texture sensitivities emerged as the most commonly reported sensory factors influencing feeding (Crasta et al. 2014; Crippa et al. 2022; Dovey et al. 2019; Johnson et al. 2014; Riccio et al. 2018; Smith et al. 2020; Zobel‐Lachiusa et al. 2015).

Three studies (Calisan Kinter et al. 2024; Chen et al. 2024; St. John and Ausderau 2024) emphasised sensory processing as central to feeding challenges in children with ARFID. Chen et al. (2024) found sensory patterns mediated the link between autistic traits and eating behaviours; Calisan Kinter et al. (2024) and St. John and Ausderau (2024) identified oral sensory sensitivity as a strong ARFID predictor, with early issues leading to worsening challenges. Cui et al. (2024) linked sensory sensitivity to nutrient deficiencies in children with autism with intellectual disabilities. Three studies (da Silva and Gomes 2024; Li et al. 2024; Sanchez‐Cerezo et al. 2024) found taste/smell sensitivities related to reduced fruit/vegetable intake, food avoidance and nutritional imbalances.

#### 3.6.3 Food Refusal and Feeding Difficulties

Twenty‐four studies examined food refusal, restrictive eating and broader feeding difficulties. Unlike food selectivity, which reflects a limited range of accepted foods, food refusal refers to the rejection of specific foods or meals. Across studies involving children with autism only, food refusal was consistently identified as one of the most common caregiver concerns, with prevalence estimates ranging from approximately 40% of foods refused to over three‐quarters of children experiencing regular food refusal (Attlee et al. 2015; Leader et al. 2021). Greater food refusal was associated with higher levels of food selectivity, lower dietary variety and increased sensory sensitivities (Chen et al. 2024; Crasta et al. 2014; Malhi et al. 2017; Tanner and Andreone 2015).

Studies including comparison groups consistently demonstrated that children with autism experienced significantly higher rates of food refusal and mealtime difficulties than neurotypical children. These included greater rejection of foods based on texture, taste, smell, food mixtures, brand and shape, together with higher levels of disruptive mealtime behaviours and feeding difficulties (Curtin et al. 2015; Dovey et al. 2019; Hubbard et al. 2014). Greater autism severity, caregiver feeding styles and sensory processing differences were also associated with more pronounced feeding problems (Aponte and Romanczyk 2016; Carter Leno et al. 2022; Chen et al. 2024; Crippa et al. 2022; Kang et al. 2022).

Several studies identified contextual and demographic influences on feeding difficulties. Fernandez et al. (2024) found 61.74% of Filipino children with autism exhibited picky eating, with early feeding issues linked to later food refusal. Alharbi (2024) reported that food refusal and rigid eating patterns worsened during the COVID‐19 pandemic. Calisan Kinter et al. (2024) found children with autism with ARFID had higher refusal and lower appetite scores than those without. Matthews et al. (2024) noted greater food fussiness and emotional undereating in autism families. Guller and Yaylaci (2024) reported widespread concerns (46%–89%). Önal and Uçar (2024) found sex‐based differences, and Nakaoka et al. (2024) validated the ASD‐MBQ in identifying feeding challenges.

Longitudinal and comparative studies revealed developmental patterns and clinical overlaps. St. John and Ausderau (2024) found early feeding difficulties, particularly with transitioning to table foods, predicted later food refusal and sensory issues. Postorino et al. (2017) identified similarities between autism and Anorexia Nervosa in symptomatology and cognitive profiles.

#### 3.6.4 Nutritional Status and Weight

Eighteen studies examined nutritional status, dietary adequacy and weight‐related outcomes among children with autism. Across studies involving children with autism only, nutritional deficiencies were commonly reported, particularly for calcium, vitamin D, vitamin B12, riboflavin, fibre and vitamin A (Graf‐Myles et al. 2013; Gray et al. 2022; Liu et al. 2016). Reduced dietary diversity and restricted food intake were frequently associated with lower nutrient intake and increased risk of micronutrient inadequate intake, particularly among children demonstrating greater food selectivity or sensory sensitivities (Cui et al. 2024; da Silva and Gomes 2024; Hümeyra İslamoğlu et al. 2024; Islam et al. 2024). Several studies also reported inadequate intake in essential fatty acids including omega‐3 and omega‐6, alongside lower consumption of fruits and vegetables compared to children of the same age (Rababah et al. 2024).

Across studies comparing children with autism and neurotypical children, children with autism generally demonstrated poorer overall nutritional intake and lower diet quality. However, the relationship between feeding behaviours and weight status was less consistent. Although several studies reported increased rates of overweight and obesity among children with autism (Islam et al. 2024; Önal and Uçar 2024; Van Der Lubbe et al. 2024), others found no significant association between food selectivity and growth, anthropometric measures or overall nutrient intake (Wenzell et al. 2024). Greater autism severity was consistently associated with poorer nutritional intake and more severe feeding difficulties (Gray et al. 2022; Johnson et al. 2014; Liu et al. 2016; Molina‐López et al. 2021; Sharp et al. 2018), suggesting that nutritional risk may be greatest among children experiencing more pronounced feeding challenges.

Several studies also highlighted parental concern regarding children's nutritional adequacy and eating behaviours. Parents frequently reported worries about dietary quality, nutritional deficiencies and weight management, particularly where feeding difficulties were persistent or highly restrictive (Sharp et al. 2018; Tahech et al. 2023).

#### 3.6.5 Feeding and Demographics

Twelve studies examined demographic factors associated with feeding behaviours. Across studies, younger age was consistently associated with greater food selectivity, food refusal and nutritional inadequacies, whereas older children generally consumed a wider variety of foods (Adams et al. 2021; Farag et al. 2022; Zickgraf and Mayes 2018). Longitudinal evidence suggested that feeding difficulties often emerged during infancy or early childhood and frequently persisted over time (Fernandez et al. 2024; St. John and Ausderau 2024).

Several studies also reported associations between autism severity and feeding behaviours, with children with autism demonstrating more severe autistic characteristics, experiencing greater food selectivity, more restricted diets, and increased feeding difficulties (Adams et al. 2021; Zickgraf and Mayes 2018). Sex differences were less consistently reported. Some studies reported higher obesity in boys, whereas girls demonstrated higher levels of broader feeding difficulties, encompassing mealtime and feeding‐related challenges rather than greater food selectivity. Boys also showed more rigid eating, and younger children had greater food selectivity (Alharbi 2024; Önal and Uçar 2024). Overall, demographic factors appeared to influence the presentation and severity of feeding difficulties, although findings relating to sex were inconsistent across studies.

#### 3.6.6 Feeding and the Environment

Six studies investigated environmental influences on feeding behaviours. Across these studies, family routines, caregiver practices, household characteristics and broader environmental factors were all found to influence children's eating behaviours and mealtime experiences. Although family environment alone did not fully explain feeding difficulties, caregiver responses and mealtime routines appeared to shape how feeding challenges were managed (Aponte and Romanczyk 2016; Thullen and Bonsall 2017).

Several studies highlighted the importance of structured home environments. Consistent mealtime routines, caregiver‐led feeding practices and supportive family environments were associated with improved feeding skills, greater parental confidence and reduced mealtime difficulties among children with autism (Chen et al. 2024; Hladik et al. 2025). Conversely, household disruption, parental stress and changes to daily routines, including those experienced during the COVID‐19 pandemic, were associated with increased food selectivity, altered eating behaviours and more challenging mealtimes among children with autism (Alharbi 2024).

Broader household factors, including sleep, parental health, household composition, smoking and participation in organised activities, were also associated with weight status and feeding outcomes among children with autism (Kim and Kwon 2022), suggesting that feeding behaviours are influenced by multiple interacting environmental and family‐level factors.

#### 3.6.7 Food Interventions

Ten studies evaluated interventions designed to improve feeding behaviours among children with autism. Although intervention approaches varied considerably, all studies reported improvements in at least one feeding‐related outcome. Common intervention strategies included structured food choice, gradual food exposure, caregiver training, sensory‐based approaches, reinforcement techniques and nutrition education.

Across intervention studies, improvements were reported in food acceptance, dietary variety, self‐feeding skills, reduced food refusal and broader food repertoires among children with autism (Cihon et al. 2020; Crowley et al. 2020; Demchuk et al. 2024; Khatoon and Senapati 2024; Sharp et al. 2013, 2014; Tanner and Andreone 2015). Several interventions also resulted in reduced caregiver stress and increased parental confidence in managing feeding behaviours (Sharp et al. 2013, 2014; Son and Alford 2024).

Despite these encouraging findings, intervention studies were generally characterised by relatively small sample sizes and diverse intervention approaches, limiting direct comparisons between studies. Nevertheless, the available evidence suggests that behavioural, sensory‐based and family‐centred interventions may all contribute to improvements in feeding behaviours and mealtime experiences for children with autism.

### 3.7 Content Analysis

Eleven qualitative and mixed‐methods studies were analysed using content analysis, identifying 10 key codes: (1) Food selectivity and refusal; (2) mother as organiser of meals; (3) desire for good nutrition; (4) mealtimes as meaningful events; (5) strategising and adapting feeding approaches; (6) stress related to feeding; (7) emotions during mealtimes (joy, stress, intimacy, avoidance); (8) challenging behaviours at meals; (9) parental desire for knowledge and gaps in understanding and (10) social influence and learning around food and autism. These themes reflect parents' experiences managing feeding challenges and highlight the emotional, social and practical dimensions of mealtimes.

In several studies (Ausderau and Juarez 2013; Curtiss and Ebata 2021; Dhillon‐Burrows et al. 2023; Gray et al. 2022; Ismail et al. 2020), parents, especially mothers, showed strong commitment to managing mealtime challenges for children with autism, recognising nutrition's importance despite obstacles. Food selectivity and refusal were common (Ausderau and Juarez 2013; Curtiss and Ebata 2021; Dhillon‐Burrows et al. 2023; Gray et al. 2022; Ismail et al. 2020; Lázaro and Pondé 2017; Suarez and Crinion 2015), making it difficult to ensure balanced diets. Parents reported stress related to feeding (Curtiss and Ebata 2019; Dhillon‐Burrows et al. 2023; Ismail et al. 2020) and used strategies like negotiation, portion control, acceptance and bulk‐buying preferred foods. Urban settings (Dhillon‐Burrows et al. 2023; Ismail et al. 2020) often led to reliance on processed foods, adding complexity. Mealtimes were seen as significant (Ausderau and Juarez 2013; Dhillon‐Burrows et al. 2023; Gray et al. 2022), particularly during lockdowns, when they became key moments for socialisation. Many parents expressed a need for more knowledge and support around nutrition and feeding strategies (Ausderau and Juarez 2013; Curtiss and Ebata 2019; Gray et al. 2022; Ismail et al. 2020), highlighting the importance of tailored interventions.

## 4 Discussion and Implications

This systematic review provides a comprehensive synthesis of the literature on feeding behaviours in children and young people with autism. Findings highlight the high prevalence of food refusal, food selectivity and sensory‐related eating challenges in this population, alongside the wider nutritional, developmental and psychosocial implications for both children and their families.

The narrative synthesis provided comprehensive insights into various feeding behaviours, nutritional status and socio‐environmental factors among children with autism. Food selectivity emerged as a predominant feeding challenge, with several studies reporting a preference for starchy foods and snacks over other food groups among children with autism, a trend also noted in the wider literature (Bandini et al. 2010; Esposito et al. 2023; Larner 2016; Moldovan‐Grunfeld 2023). A recent systematic review (Rodrigues et al. 2023) which compared children with autism to neurotypical children found that those with autism tended to exhibit higher levels of food selectivity and neophobia. This was characterised by a limited variety of foods consumed, strong preferences for specific textures, colours or brands of foods and a fear of trying new foods compared to neurotypical children. Consistent with these findings, our review revealed that children with autism were often influenced by food presentation, specific brands and packaging. Common mealtime behaviours such as ‘fussiness’ and the need for diversion during meals were also observed. Additionally, studies indicated a high prevalence of food refusal and feeding difficulties among children with autism, often associated with texture, taste and sensory hypersensitivity.

The findings also indicate that children with food selectivity demonstrate significantly higher rates of autism traits, reluctance to eat novel foods and selectivity regarding the range of foods they accept. This links to wider research linked to the gut‐brain axis and the implications of food insecurity in this context. The gut‐brain axis refers to the bidirectional communication between the central nervous system and the gastrointestinal tract, highlighting the intricate relationship between gut health and neurological function (Rutsch et al. 2020). Food selectivity, often observed in children with autism, may lead to nutritional deficiencies, impacting gut health and consequently exacerbating autism traits (Demissew Beyene 2023). Therefore, addressing food selectivity appears essential not only for ensuring adequate nutrition but also for supporting function and mitigating the severity of autism traits.

The finding that food selectivity among children with autism is not associated with compromised growth or obesity prompts further inquiry into the relationship between food selectivity and nutritional outcomes (Esposito et al. 2023). Although growth may appear unaffected, children with severe food selectivity may still experience nutritional inadequate intake or other health challenges that are not reflected in weight or height measures alone. Future research could help clarify these nuances, identify protective or moderating factors, and provide a more comprehensive understanding of the nutritional needs and growth trajectories of this population.

Despite the prevalence of food selectivity, some children with autism showed occasional willingness to try new foods. Older children tended to consume a greater variety of foods, but autism severity was inversely correlated with the number of food items eaten. This highlights the significant impact of autism severity on dietary diversity, with individuals experiencing more severe traits having more restricted diets. Therefore, targeted support and interventions are essential for individuals with autism who face greater challenges in maintaining a varied diet. Given the complexity of this issue, it is crucial to develop and implement tailored support strategies that address the specific needs of the individual, ensuring they receive adequate nutrition for optimal health and well‐being (Bandini et al. 2010).

Environmental factors, such as household structure, urban environments, parental health and participation in organised activities, were found to have a significant impact on eating behaviours and weight‐related concerns among children with autism. This finding is consistent with broader literature indicating the influence of environmental factors on this population's health and well‐being (Curtin et al. 2014; Mazurek and Kanne 2010). The family environment in particular appears to play a crucial role in shaping dietary habits and weight‐related concerns in this population (Cermak et al. 2010). Additionally, household routines, mealtime practices and parental modelling of healthy eating behaviours have been identified as important determinants of dietary intake and weight status (Schreck and Williams 2006). Furthermore, feeding interventions have demonstrated positive outcomes in addressing feeding difficulties and improving eating behaviours among children with autism. Interventions focusing on sensory‐based feeding approaches, structured mealtime routines and behavioural strategies seem to be effective in increasing food acceptance and reducing food refusal behaviours in this population (Bandini et al. 2010). These findings are consistent with previous research indicating the efficacy of targeted feeding interventions in improving feeding behaviours and nutritional outcomes among children with autism (Lukens and Linscheid 2008).

Although food selectivity and food refusal are well‐established features of feeding behaviours in children with autism, the findings of this review suggest that these behaviours are unlikely to arise from sensory sensitivities alone. Rather, they appear to reflect a dynamic interaction between child characteristics, family circumstances and the wider environment. Sensory processing differences were consistently associated with greater food selectivity, food refusal and atypical eating behaviours, whereas greater autism severity was frequently linked with more pronounced feeding difficulties (Adams et al. 2021; Aponte and Romanczyk 2016; Crippa et al. 2022; Mayes and Zickgraf 2019). However, environmental factors also appeared to play an important role. Feeding behaviours reportedly worsened during the COVID‐19 pandemic, with increased food refusal and rigid eating patterns observed during this period of disruption (Alharbi 2024). Similarly, studies examining mealtime environments reported that household stress, family chaos and mealtime disruption were associated with greater food fussiness, emotional undereating and feeding challenges (Matthews et al. 2024). Collectively, these findings suggest that feeding behaviours may be reinforced during periods of uncertainty or heightened stress, where changes in routine, anxiety and reduced predictability interact with underlying sensory sensitivities.

The findings also suggest that feeding difficulties should be viewed within a broader ecological context. Families managing highly selective diets may experience increased financial pressures through restricted food choices, food waste, and the need to purchase specific preferred foods. These practical challenges may reduce the time, financial resources and emotional capacity available to implement feeding interventions consistently. Previous research has also demonstrated that families of children with autism experience higher rates of food insecurity than the general population (Karpur et al. 2021), and the current review identified poorer dietary diversity and nutritional inadequacies among children with greater food selectivity (Cui et al. 2024; Wenzell et al. 2024). Together, these findings suggest that feeding behaviours represent a complex systems issue requiring coordinated support from dietitians, psychologists, occupational therapists and wider family support services, alongside policies that address food insecurity and improve access to affordable, nutritionally appropriate foods.

The content analysis revealed 10 recurring codes related to feeding behaviours and mealtimes of children with autism. Across these studies, parents, particularly mothers, demonstrated a strong commitment to understanding and addressing the challenges associated with mealtimes for their children. Despite facing numerous obstacles, parents recognised the importance of nutrition and strove to provide healthy meals. Food selectivity and refusal were prevalent among children with autism, posing significant challenges for parents in ensuring a balanced diet. Parents reported experiencing stress related to their children's dietary habits and employed various coping mechanisms including negotiation and portion size control. However, there was a desire among the parents for more knowledge and support regarding nutrition and mealtime strategies, indicating the need for tailored interventions and educational resources (Vohra et al. 2013).

The emphasis on the significance of mealtimes, particularly during lockdown conditions, highlighted the increased attention and opportunities for socialisation observed in many households. However, for children with autism facing food selectivity or food insecurity, mealtimes presented substantial challenges. This brings to light the complex nature of mealtimes, serving not only as occasions for nutrition but also as critical opportunities for socialisation. Consequently, if a child has food issues or food insecurity, a fundamental premise of food as a social activity is diminished. This disruption in the social aspect of mealtimes may have long‐term impacts on the development of social skills and overall well‐being. A recent study (Blake and Cromwell 2022) looking at food insecurity within UK communities found that food insecurity increases stress, depression and feelings of social isolation. They found that social eating, for example breakfast clubs, after school clubs and soup kitchens, offered many benefits including mental health support, befriending groups and building social ties. Exploring the links between food issues, food insecurity and the social dynamics of mealtimes, as well as their potential long‐term consequences, presents an essential avenue for future research.

At last, an important aspect highlighted in this review is the outdated terminology used in some of the included papers to describe the eating habits of children with autism. Terms such as ‘fussy’ or ‘picky‐eaters’ were found to be prevalent in the literature. However, it is important to recognise that these terms carry a negative connotation and can perpetuate misconceptions about the eating habits of children with autism. Using such terminology may undermine the significance of the challenges faced by these children and their families. Instead, it is advised to use more accurate and respectful language that reflects the complex nature of these eating behaviours. Research has shown that selective eating habits often extend beyond mere ‘fussiness’ and can be influenced by sensory sensitivities, food aversions and other factors associated with autism (Rastam 2008). Therefore, employing more neutral and descriptive terminology is essential to foster a better understanding of the unique feeding challenges experienced by children with autism.

## 5 Study Limitations

The review included few qualitative studies despite extensive database searches. This limited the ability to capture the full complexity of feeding experiences in families with autism. Although quantitative data offered valuable insights (albeit not in a way that allowed us to compute general effects), the lack of qualitative research restricted the depth and contextual richness of the findings. Additionally, the studies varied widely in design, outcome measures, and tools used to assess feeding behaviours and nutritional intake. This variability limited the ability to conduct a meta‐analysis and may reduce the comparability of findings across studies. At last, many studies relied primarily on parent or caregiver reports, which are subject to recall bias and social desirability bias. This may lead to under‐ or over‐reporting of feeding difficulties and dietary intake.

These limitations may have resulted in either underestimation or overestimation of feeding difficulties. Reliance on parent‐reported outcomes may have introduced reporting bias, whereas heterogeneity in outcome measures likely reduced comparability across studies and limited the ability to identify precise effect estimates.

## 6 Conclusions

This systematic review demonstrates that feeding difficulties are a common and clinically significant issue among children and young people with ASD. Across 88 studies, food selectivity, food refusal, sensory‐related feeding challenges and restricted dietary variety emerged as the most consistently reported feeding difficulties. Sensory sensitivities, particularly those related to taste, smell and texture, were repeatedly associated with feeding problems and appear to represent a key mechanism underlying many eating‐related challenges. Although nutritional and weight‐related outcomes varied across studies, there was consistent evidence of reduced dietary diversity and increased risk of nutritional inadequacies among some groups of autistic children. The findings further demonstrate that feeding difficulties extend beyond nutrition alone, affecting family functioning, parental stress and mealtime experiences. Collectively, the evidence supports the need for routine assessment of feeding difficulties in autistic children and highlights the potential value of early, targeted and family‐centred interventions.

This review also highlights that mealtimes for children with autism are not solely nutritional events, but carry important social significance. Disruptions caused by feeding difficulties may hinder the development of social skills and affect overall well‐being. Moreover, when food insecurity is present, it can further intensify these challenges by limiting food availability, increasing parental stress, and reducing opportunities for shared, positive mealtime experiences. As such, further research is needed to explore how feeding behaviours and food insecurity together influence social interactions during mealtimes and their potential long‐term effects. Finally, the findings reinforce the importance of targeted support strategies for children with autism and their families, particularly in addressing persistent food refusal.

## Author Contributions

E.T. methodology, literature search, screening, data extraction, quality appraisal, formal analysis, data curation, writing – original draft, writing – review and editing. J.S. methodology, supervision, writing – review and editing. B.H. screening, data extraction, quality appraisal, writing – review and editing. M.Y. methodology, supervision, writing – review and editing. L.C. supervision, writing – review and editing. E.L.G. conceptualisation, methodology, supervision, writing – review and editing.

## Funding

The authors have nothing to report.

## Conflicts of Interest

The authors declare no conflicts of interest.

## Supporting information


**File S1:** Search terms.

**File S2:** nbu70066‐sup‐0002‐FileS2.xlsx.

**File S3:** nbu70066‐sup‐0003‐FileS3.xlsx.

**Table S1:** Tool characteristics.

## Data Availability

The data that support the findings of this study are available from the corresponding author upon reasonable request.
