# Supplementary material for: Feeding Behaviours in Families With Children or Young People With Autism: A Systematic Review
Source: Nutr Bull. 2026 Aug 18;51(3):285–321. doi: 10.1111/nbu.70066 (PMC13522876; doi:10.1111/nbu.70066)
Supplement: Supplementary file 1 [file NBU-51-285-s001.docx]

**Supplementary File S1. Search Terms**

| **P**opulation | Autistic children and young people below the age of 25 years old and their family members | “autism” OR “autistic” OR “ASD” OR “autism spectrum disorder” OR “autism spectrum” OR “autistic disorder” AND “children” OR “young people” OR “young” OR “child*” OR “kid*” OR “youth*” OR “minor*” OR “infant*” AND “family” OR “family member*” OR “mother” OR “mum” OR “mam” OR “mom” OR “father” OR “dad” OR “siblings” OR “brother” OR “sister” |
| --- | --- | --- |
| **I**ntervention/exposure | Food insecurity and/or feeding behaviours | “Food insecur*” OR “Food poverty” OR “Food deprivation” OR “Food insufficiency” OR “lack of food” OR “Food hardship”  “Feeding behavio*” OR “eating habits” OR “dietry patterns” OR “nutrition habits” OR “food intake” OR “problem feeding” OR “eating behavio*” OR “mealtime behavio*” OR “feeding patterns” OR “dietary behavio*” OR “food consumption” |
| **C**omparators | Typically developed children | “typically developed children” OR “TDC” OR “non-ASD” OR “non-autistic” OR “typically developed” OR “TD” |
| **O**utcomes | Experiences of food insecurity and/or feeding behaviours described in cross-sectional, cohort, or quantitative research | “experience*” OR “cross-sectional” OR “cohort” OR “quantitative” OR “qualitative” OR “intervention” |
