# Supplementary material for: Feeding Behaviours in Families With Children or Young People With Autism: A Systematic Review
Source: Nutr Bull. 2026 Aug 18;51(3):285–321. doi: 10.1111/nbu.70066 (PMC13522876; doi:10.1111/nbu.70066)
Supplement: Supplementary file 2 [file NBU-51-285-s004.docx]

**Supplementary Table S1: Tool Characteristics**

| Assessment type | Formally Validated? | Validated Specifically for Autistic Children? | Primary Target Population /Measures dietary intake (Y/N) |
| --- | --- | --- | --- |
| Formally Validated Measures |  |  |  |
| BAMBI (*Brief Autism Mealtime Behaviour Inventory*) | Yes | Yes | Autistic children (ages 3–11). Designed specifically for ASD core mealtime issues / N |
| BAMBIC (*Brief Assessment of Mealtime Behaviours in Children*) | Yes | Yes (Evaluated) | Adapted from BAMBI for general paediatric population; validated across typical and autistic cohorts / N |
| BPFAS (*Behavioural Paediatric Feeding Assessment Scale*) | Yes | Yes (Evaluated) | General paediatric population; extensively evaluated and validated in autistic children / N |
| CEBQ (*Child Eating Behavior Questionnaire*) | Yes | Yes (Evaluated) | General paediatric population (ages 2–9 years); validated/evaluated in autism studies / N |
| CEBI (*Children's Eating Behavior Inventory*) | Yes | Yes (Evaluated) | Paediatric populations with/without special needs, including ASD / N |
| CFQ (*Child Feeding Questionnaire*) | Yes | Yes (Evaluated) | Measures parent feeding practices; evaluated in paediatric ASD cohorts / N |
| CFNS (*Child Food Neophobia Scale*) | Yes | Yes (Evaluated) | General paediatric population; evaluated in autistic children / N |
| CFSQ (*Caregiver Feeding Style Questionnaire*) | Yes | No (General Paediatric) | Validated for general paediatric caregiver feeding styles / N |
| EAT-26 (*Eating Attitudes Test-26*) | Yes | No (General/Adolescents) | Standardised eating disorder screen; validated in adolescents/adults, not young/autistic children / N |
| EDI-3 (*Eating Disorder Inventory-3*) | Yes | No (General/Adolescents) | Standardized ED inventory for older youth (13+); not designed/validated for childhood ASD / N |
| FEAST (*Feeding and Eating in AutiSm Together*) | Yes | Yes | Autistic children (ages 2–12). Developed specifically for ASD feeding profiles / N |
| FFQ / YAQ (*Food Frequency Questionnaire / Youth-Adolescent FFQ*) | Yes | Yes (Evaluated) | Epidemiological dietary intake tools; validated in general paediatrics and evaluated in ASD / Y |
| FPQ (*Food Preference Questionnaire*) | Yes | Yes (Evaluated) | Validated in general paediatrics; frequently adapted/evaluated for picky eating & ASD / N |
| French AFNS (*Adapted Food Neophobia Scale*) | Yes | No (General Paediatric) | French adaptation validated for general paediatric neophobia / N |
| Labyrinth Scale (*Escala Labirinto*) | Yes | Yes (Brazil) | Validated in Brazil specifically as a diagnostic/behavioural tool for autistic children (ages 2–5) / N |
| PediEAT (*Pediatric Eating Assessment Tool*) | Yes | Yes (Evaluated) | General pediatric population (6 mos – 7 yrs); validated and evaluated across ASD cohorts / N |
| PFSQ (*Parental Feeding Style Questionnaire*) | Yes | No (General Paediatric) | Validated for parent feeding practices in general paediatrics / N |
| RAISD (*Reinforcer Assessment for Individuals with Severe Disabilities*) | Yes | Yes | Validated clinical preference assessment tool for intellectual and developmental disabilities (including ASD) / N |
| SPCQ / SSP (*Sensory Profile Caregiver Questionnaire / Short Sensory Profile*) | Yes | Yes | General sensory processing measures; extensive psychometric validation in autistic children / N |
| STEP-CHILD (*Screening Tool of Feeding Problems for Children*) | Yes | Yes | Validated paediatric feeding screening tool; widely evaluated in autistic samples / N |
| SWEAA (*Swedish Eating Assessment for ASD*) | Yes | Yes | Developed and validated specifically for adolescents/adults with ASD (less suitable for young children) / N |
| Unvalidated / Descriptive / Non-Assessment Tools |  |  |  |
| Adaptation of the Mealtime Survey | No | No | Ad-hoc adaptation derived from Provost et al., (2010); lacks formal psychometric validation / N |
| FPI (*Food Preference Inventory*) | No | No | Unstandardised descriptive checklist of food items/likes/dislikes / N |
| MIOH (*Meals in Our Household Questionnaire*) | Partial / Limited | No | Measures family mealtime environment; lacks full psychometric validation for child feeding / N |
| Survey for Characterization of Feeding Challenges... (US) | No | No | A cross-sectional epidemiological survey tool |
| USDA MyPlate | N/A | N/A | Not an assessment tool. It is a visual public-health dietary guide / N |
| Researcher-developed questionnaires (n = 8) | No | No | Study-specific questionnaires developed or adapted by the research teams; content and psychometric testing varied across studies / N |
